# Characterization of the Brain Functional Architecture of Psychostimulant Withdrawal Using Single-Cell Whole-Brain Imaging

**DOI:** 10.1523/ENEURO.0208-19.2021

**Published:** 2021-11-02

**Authors:** Adam Kimbrough, Marsida Kallupi, Lauren C. Smith, Sierra Simpson, Andres Collazo, Olivier George

**Affiliations:** 1 School of Medicine, Department of Psychiatry, University of California San Diego, La Jolla, CA 92093; 2 College of Veterinary Medicine, Department of Basic Medical Sciences, Purdue University, West Lafayette, IN 47907; 3 Department of Neuroscience, The Scripps Research Institute, La Jolla, CA 92037; 4 Beckman Institute, Cal-Tech, Pasadena, CA 91125

**Keywords:** addiction, functional connectivity, graph theory, iDISCO, neural activity, withdrawal

## Abstract

Numerous brain regions have been identified as contributing to withdrawal behaviors, but it is unclear the way in which these brain regions as a whole lead to withdrawal. The search for a final common brain pathway that is involved in withdrawal remains elusive. To address this question, we implanted osmotic minipumps containing either saline, nicotine (24 mg/kg/d), cocaine (60 mg/kg/d), or methamphetamine (4 mg/kg/d) for one week in male C57BL/6J mice. After one week, the minipumps were removed and brains collected 8 h (saline, nicotine, and cocaine) or 12 h (methamphetamine) after removal. We then performed single-cell whole-brain imaging of neural activity during the withdrawal period when brains were collected. We used hierarchical clustering and graph theory to identify similarities and differences in brain functional architecture. Although methamphetamine and cocaine shared some network similarities, the main common neuroadaptation between these psychostimulant drugs was a dramatic decrease in modularity, with a shift from a cortical-driven to subcortical-driven network, including a decrease in total hub brain regions. These results demonstrate that psychostimulant withdrawal produces the drug-dependent remodeling of functional architecture of the brain and suggest that the decreased modularity of brain functional networks and not a specific set of brain regions may represent the final common pathway associated with withdrawal.

## Significance Statement

A key aspect of treating drug abuse is understanding similarities and differences of how drugs of abuse affect the brain. In the present study, we examined how the brain is altered during withdrawal from psychostimulants. We found that each drug produced a unique pattern of activity in the brain, but that brains in withdrawal from cocaine and methamphetamine shared similar features. Interestingly, we found the major common link between withdrawal from all psychostimulants, when compared with controls, was a shift in the broad organization of the brain in the form of reduced modularity. Reduced modularity has been shown in several brain disorders, including traumatic brain injury, and dementia, and may be the common link between drugs of abuse.

## Introduction

Psychostimulants are a class of highly addictive and commonly abused drugs that includes cocaine, nicotine, and methamphetamine (Balfour, 2008; Phillips et al., 2014). A large number of brain regions have been implicated in withdrawal associated with psychostimulant use (Kalivas and McFarland, 2003; Robinson and Kolb, 2004; Kalivas, 2007; Everitt et al., 2008; Jedynak et al., 2012; Koob and Volkow, 2016; Bobadilla et al., 2017). However, the complete neural network that is associated with psychostimulant withdrawal remains understudied, and the search for a common brain pathway that is responsible for psychostimulant withdrawal remains elusive. Common features of withdrawal may not be found at the brain region level but rather at the network level.

The identification of changes in neural network structure that are caused by psychostimulant withdrawal may be critical to understanding the ways in which these drugs affect the brain. Previous studies identified changes in network function after psychostimulant use (Tomasi et al., 2010; Konova et al., 2013, 2015; Ma et al., 2015), but these analyses focused on macroscale changes and not the mesoscale level, or they focused on preselected regions of interest.

The present study sought to identify the ways in which withdrawal from different commonly abused psychostimulants alters functional architecture of the brain. We hypothesized that withdrawal from psychostimulants would result in changes in functional neural networks and decrease modular structuring of the brain. We further hypothesized that each psychostimulant that was examined herein (i.e., methamphetamine, nicotine, and cocaine) would have a unique neural network that is associated with withdrawal. We measured single-cell whole-brain activity using Fos as a marker for neuronal activation in mice that underwent withdrawal from chronic psychostimulant (cocaine, methamphetamine, and nicotine) administration. To accomplish this, mice were implanted with osmotic minipumps for one week to induce dependence to each drug. Following one-week minipumps were removed and brains were collected from mice during acute withdrawal. This method of acute withdrawal was chosen to control the amount of drug each animal received and create strong dependence in a short period of time. The psychostimulant doses were chosen based on previous studies that reported rewarding effects during use and observed withdrawal-like symptoms after the cessation of chronic exposure for each drug (Johnson et al., 2008; Fish et al., 2010; Eisener-Dorman et al., 2011; Stoker and Markou, 2011; Stoker et al., 2012; Tracy et al., 2016; Zhu et al., 2017). We then used single-cell whole-brain activity to identify coactivation patterns of brain regions in the network that was associated with each treatment using hierarchical clustering. The functional connectivity measures were used to determine the modular structuring of each network. Graph theory was then used to further characterize each network to determine the brain regions that are most heavily involved in intramodular and intermodular connectivity of the functional network.

## Materials and Methods

### Animals

Male C57BL/6J mice were bred at The Scripps Research Institute. They were 20–30 g and 60 d old at the start of the experiment. The mice were maintained on a 12/12 h light/dark cycle with *ad libitum* access to food and water. All of the procedures were conducted in strict adherence to the National Institutes of Health *Guide for the Care and Use of Laboratory Animals* and approved by The Scripps Research Institute Institutional Animal Care and Use Committee and by the Institutional Animal Care and Use Committee of the University of California.

### Drugs

The doses were 4 mg/kg/d for methamphetamine, 24 mg/kg/d for nicotine, and 60 mg/kg/d for cocaine. These doses were chosen based on previous studies that indicated rewarding effects during use, resulting in withdrawal-like symptoms after the cessation of chronic use (Johnson et al., 2008; Fish et al., 2010; Eisener-Dorman et al., 2011; Stoker and Markou, 2011; Stoker et al., 2012; Tracy et al., 2016; Zhu et al., 2017). Each drug was dissolved in saline, and the pH was adjusted to 7.4. The drugs were loaded into osmotic minipumps **(**Alzet; model 1002**)**. The minipumps sat overnight in saline before insertion to ensure that drug delivery would begin immediately.

### Minipump implantation and removal

The mice were split into four groups for the experiment: methamphetamine withdrawal group (*n *=* *5), nicotine withdrawal group (*n *=* *5), cocaine withdrawal group (*n *=* *5), and saline control group (*n *=* *4). Each mouse was surgically implanted with an osmotic minipump for methamphetamine, nicotine, cocaine, and saline based on group assignment. The minipumps were implanted in the lower back of each mouse under anesthesia. After brief recovery, the mice were returned to their home cages. The mice remained in their home cages for one week to allow for chronic infusion of the drug.

After one week, the minipumps were surgically removed under anesthesia to allow for drug washout and withdrawal to begin. Mice in the nicotine, cocaine, and saline groups were perfused 8 h after removal of the minipumps. Mice in the methamphetamine group were perfused 12 h after removal of the minipumps. These time points were chosen to represent an acute withdrawal period from each drug (e.g., a minimum of 4 h without the drug present) and based on the half-life of each drug in mice (Benuck et al., 1987; Cho et al., 2001; Norman et al., 2007; Siu and Tyndale, 2007; Shabani et al., 2012).

### Tissue collection

The mice were deeply anesthetized and perfused with 15 ml of PBS followed by 50 ml of 4% formaldehyde. The brains were postfixed in formaldehyde overnight. The next day, the brains were washed for 30 min three times with PBS and transferred to a PBS/0.1% azide solution at 4°C for 2–3 d before processing via iDISCO+.

### iDISCO+

**Figure 1.**
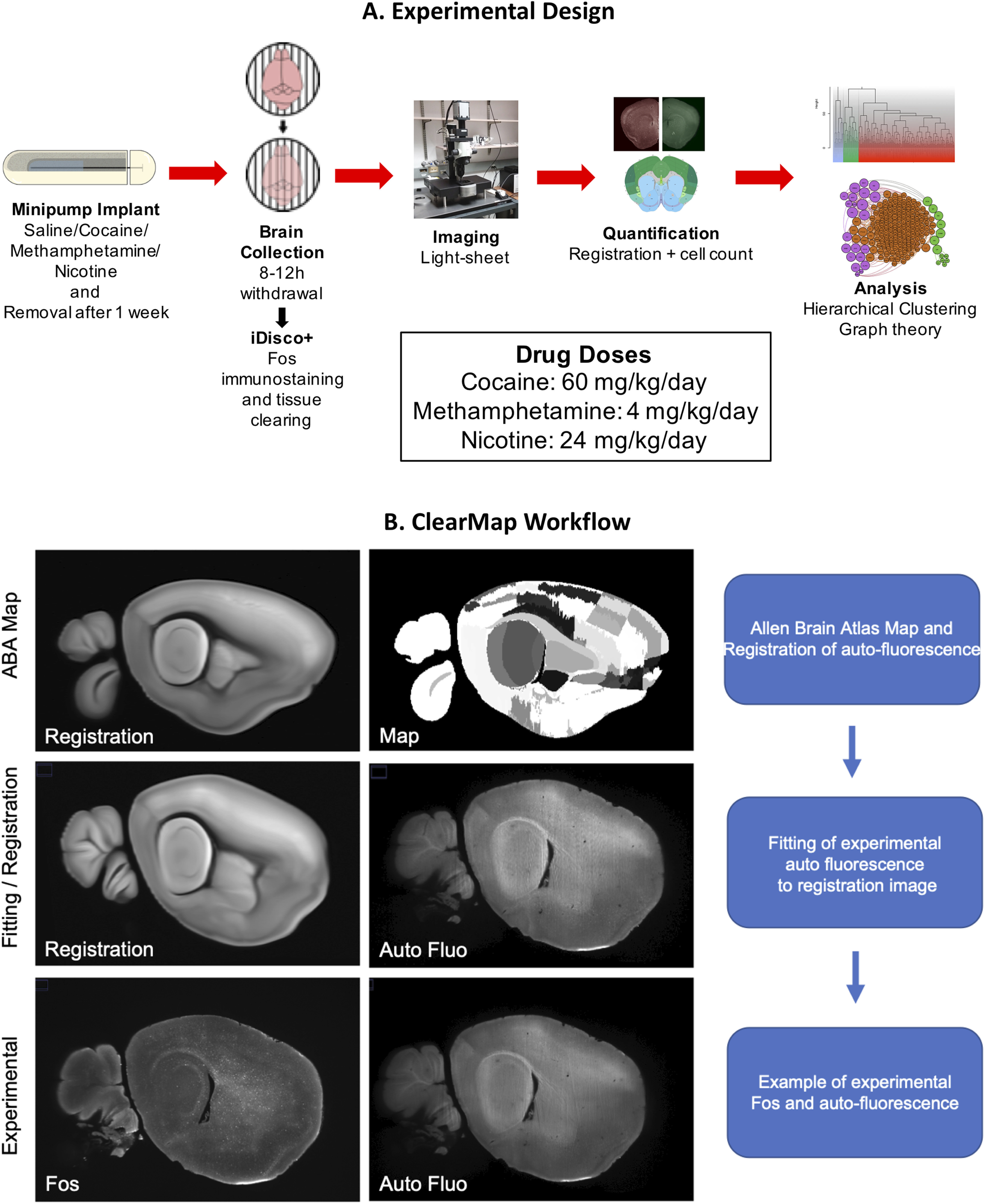
***A***, Experimental design. Mice were surgically implanted with an osmotic minipump that contained either saline or a psychostimulant (60 mg/kg/d cocaine, 4 mg/kg/d methamphetamine, or 24 mg/kg/d nicotine). They were then returned to their home cage for one week. After one week, the minipumps were surgically removed, and the mice were returned to their home cage until brain tissue was collected 8 h later (saline, cocaine, nicotine) or 12 h later (methamphetamine). Brains were then processed for whole-brain Fos immunohistochemistry and clearing via iDISCO+ and then imaged on a light-sheet microscope. Fos values were detected and registered to the Allen Brain Atlas using ClearMap Renier et al., 2016. Pearson correlations were then calculated to determine functional coactivation among brain regions. Brain regions were then grouped into modules based on their coactivation patterns through hierarchical clustering. Graph theory analyses was then performed to identify brain regions that are heavily involved in intramodular and intermodular connectivity. ***B***, Workflow diagram of registration to the Allen Brain Atlas using ClearMap. Registration is performed by matching a the autofluorescence to a preregistered two-photon image set that has been matched to brain region delineations of the Allen Brain Atlas. The brain region demarcations mapped to the autofluorescence are then used to map onto the Fos values taken from the corresponding frame. Auto Fluo = Autofluorescence.

The iDISCO+ procedure was performed as reported previously (Renier et al., 2014, 2016). The associated immunostaining, sample clearing, and image collection for iDISCO+ are detailed below. For an experimental design overview see Figure 1.

### Immunostaining

Fixed samples were washed in 20% methanol (in double-distilled H_2_O) for 1 h, 40% methanol for 1 h, 60% methanol for 1 h, 80% methanol for 1 h, and 100% methanol for 1 h twice. The samples were then precleared with overnight incubation in 33% methanol and 66% dichloromethane (DCM; Sigma, catalog #270997-12X100ML). The next day, the samples were bleached with 5% H_2_O_2_ (1 volume of 30% H_2_O_2_ for 5 volumes of methanol, ice cold) at 4°C overnight. After bleaching, the samples were slowly re-equilibrated at room temperature and rehydrated in 80% methanol in double-distilled H_2_O for 1 h, 60% methanol for 1 h, 40% methanol for 1 h, 20% methanol for 1 h, PBS for 1 h, and PBS and 0.2% Triton X-100 for 1 h twice. The samples were then incubated in PBS, 0.2% Triton X-100, 20% dimethylsulfoxide (DMSO), 0.3 m glycine at 37°C for 2 d and then blocked in PBS, 0.2% Triton X-100, 10% DMSO, and 6% donkey serum at 37°C for 2 d. The samples were then incubated in rabbit anti c-*fos* (1:2000; Synaptic Systems catalog #226003) in PBS-0.2% Tween with 10 μg, ml heparin (PTwH), and 5% DMSO/3% donkey serum at 37°C for 7 d. The samples were then washed in PTwH for 24 h (five changes of the PTwH solution over that time) and incubated in donkey anti-rabbit Alexa Fluor 647 (1:500; Invitrogen, catalog #A31573) in PTwH/3% donkey serum at 37°C for 7 d. The samples were finally washed in PTwH for 1 d before clearing and imaging.

### Sample clearing

Immunolabeled brains were cleared using the procedure of Renier et al. (2016). The samples were dehydrated in 20% methanol in double-distilled H_2_O for 1 h, 40% methanol for 1 h, 60% methanol for 1 h, 80% methanol for 1 h, 100% methanol for 1 h, and 100% methanol again overnight. The next day, the samples were incubated for 3 h in 33% methanol/66% DCM until they sank to the bottom of the incubation tube. The methanol was then washed for 20 min twice in 100% DCM. Finally, the samples were incubated in dibenzyl ether (DBE; Sigma, catalog #108014-1KG) until clear and then stored in DBE at room temperature until imaged.

### Image acquisition

**Figure 2.**
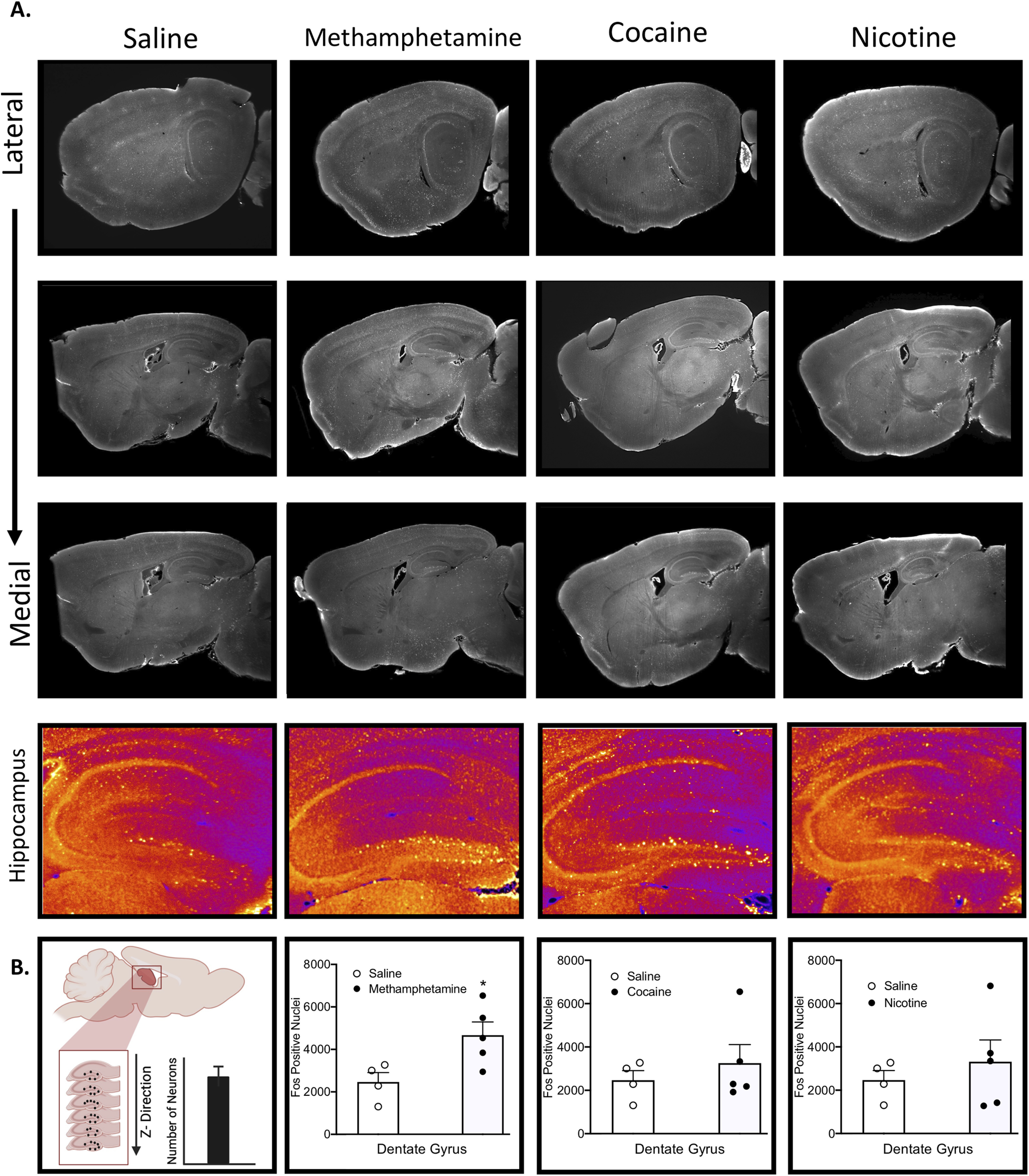
***A***, Lateral to medial sagittal representative sections of the brain and zoomed in representative hippocampal subsections for each treatment. ***B***, Comparisons of Fos values for saline versus each treatment in the dentate gyrus. See Extended Data Figure 2-2 for raw Fos values and Extended Data Figure 2-1 for comparisons of raw Fos for treatments versus saline.

Left hemispheres of cleared samples were imaged in the sagittal orientation (right lateral side up). A single hemisphere was imaged as done in previous studies to avoid the need to stitch images or analyze separate image stacks for the same sample (Renier et al., 2014, 2016). Future studies examining both hemispheres would provide interesting additional results. Samples were imaged on a light-sheet microscope (Ultramicroscope II, LaVision Biotec) equipped with an sCMOS camera (Andor Neo) and 2×/0.5 objective lens (MVPLAPO 2×) equipped with a 6-mm working distance dipping cap. Imspector Microscope controller v144 software was used. The microscope was equipped with an NKT Photonics SuperK EXTREME EXW-12 white light laser with three fixed light sheet generating lenses on each side. Scans were made at 0.8× magnification (1.6× effective magnification) with a light sheet numerical aperture of 0.148. Excitation filters of 480/30, 560/40, and 630/30 nm were used. Emission filters of 525/50, 595/40, and 680/30 nm were used. The samples were scanned with a step size of 3 μm using dynamic horizontal scanning from one side (the right) for the 560- and 630-nm channels (20 acquisitions per plane with 240-ms exposure, combined into one image using the horizontal adaptive algorithm) and without horizontal scanning for the 480-nm channel using two-sided illumination (100-ms exposure for each side, combined into one image using the blending algorithm). To accelerate acquisition, both channels where acquired in two separate scans. The imaging resolution (*x* = 4 μm, *y* = 4 μm, *z* = 3 μm) was selected to minimize imaging time without loss in terms of sensitivity or selectivity of the cell detection process or brain segmentation. The approach of clearing, alignment, cell detection, and registration has been validated in great detail in the original Renier et al. (2016) paper and shows that cell count obtained using ClearMap is 99% similar to manual detection by a trained user (Renier et al., 2016) when using a conservative cell voxel size threshold of 20 pixel (as in our study). The cell segmentation parameters and intensity threshold used to identify Fos-positive cells in this study are the default settings included in the ClearMap package (Renier et al., 2016) without further validation, but visual confirmation was made manually on every brain to verify appropriate alignment to the reference atlas and to verify that thresholding and pixel detection were set to maximize the number of cells detected while ensuring that cells were not double counted. To account for micro-movements of the samples that may occur between scans, three-dimensional image affine registration was performed to align both channels using ClearMap (Renier et al., 2016). Representative images of Fos collected can be seen in Figure 2.

10.1523/ENEURO.0208-19.2021.f2-1 Extended Data Figure 2-1 Fos counts of brain regions showing significant differences between a treatment and saline. Download Figure 2-1, TIF file.

10.1523/ENEURO.0208-19.2021.f2-2 Extended Data Figure 2-2 Table of raw Fos values and group SEMs for each treatment. Download Figure 2-2, XLS file.

### Data analysis

#### Identification of activated brain regions

Images that were acquired from the light-sheet microscope were analyzed from the end of the olfactory bulbs (the olfactory bulbs were not included in the analysis) to the beginning of the hindbrain and cerebellum. Counts of Fos-positive nuclei from each sample were identified for each brain region using ClearMap (Renier et al., 2016). ClearMap uses autofluorescence that is acquired in the 488-nm channel to align the brain to the Allen Mouse Brain Atlas (Allen Institute for Brain Science, 2004) and then registers Fos counts to regions that are annotated by the atlas. ClearMap has been validated and used now in several recent studies to identify labeled neurons and quantify the number labeled in a given brain region (Liebmann et al., 2016; Renier et al., 2016; Kimbrough et al., 2020; Kirst et al., 2020; Qian et al., 2021). For raw Fos counts and information on brain regions showing significant differences between saline and treatment Fos levels assessed by traditional comparison see the Extended Data Figures 2-1 and 2-2. A potential confound of the present approach is that possible errors in atlas registration, although unlikely, are would impact data from smaller brain regions more than larger brain regions. The data were normalized to a log_10_ value to reduce variability and bring brain regions with high numbers (e.g., thousands) and low numbers (e.g., tens to hundreds) of Fos counts to a similar scale.

#### Identification of functional connectivity within individual networks

Separate interregional Pearson correlations were then calculated using Statistica software (Tibco) across animals in the saline, cocaine, methamphetamine, and nicotine groups to compare the log_10_ Fos data from each brain region to each of the other brain regions. See Table 1 for a list of brain regions, their abbreviations, and their Allen atlas grouping. It should be noted that connectivity throughout refers to functional connectivity of brain regions and not structural connectivity.

**Table 1:** Brain regions list

Brain region	Abbreviation	Allen Group name
Agranular insular area posterior part	AIp	Cortical plate
Agranular insular area ventral part	AIv	Cortical plate
Anterior cingulate area dorsal part	ACAd	Cortical plate
Anterior cingulate area ventral part	ACAv	Cortical plate
Anterior olfactory nucleus	AON	Cortical plate
Anterolateral visual area	VISal	Cortical plate
Anteromedial visual area	VISam	Cortical plate
Cortical amygdalar area posterior part	COAp	Cortical plate
Dentate gyrus	DG	Cortical plate
Dorsal auditory area	AUDd	Cortical plate
Dorsal peduncular area	DP	Cortical plate
Ectorhinal area	ECT	Cortical plate
Entorhinal area lateral part	ENTl	Cortical plate
Entorhinal area medial part	ENTm	Cortical plate
Fasciola cinerea	FC	Cortical plate
Field CA1	CA1	Cortical plate
Field CA2	CA2	Cortical plate
Field CA3	CA3	Cortical plate
Frontal pole cerebral cortex	FRP	Cortical plate
Gustatory areas	GU	Cortical plate
Induseum griseum	IG	Cortical plate
Infralimbic area	ILA	Cortical plate
Lateral visual area	VISl	Cortical plate
Nucleus of the lateral olfactory tract	NLOT	Cortical plate
Orbital area lateral part	ORBl	Cortical plate
Orbital area medial part	ORBm	Cortical plate
Orbital area ventrolateral part	ORBvl	Cortical plate
Parasubiculum	PAR	Cortical plate
Perirhinal area	PERI	Cortical plate
Piriform area	PIR	Cortical plate
Piriform-amygdalar area	PAA	Cortical plate
Posterior auditory area	AUDpo	Cortical plate
Posterolateral visual area	VISpl	Cortical plate
Posteromedial visual area	VISpm	Cortical plate
Postpiriform transition area	TR	Cortical plate
Postsubiculum	POST	Cortical plate
Prelimbic area	PL	Cortical plate
Presubiculum	PRE	Cortical plate
Primary auditory area	AUDp	Cortical plate
Primary motor area	MOp	Cortical plate
Primary somatosensory area barrel field	SSp-bfd	Cortical plate
Primary somatosensory area lower limb	SSp-ll	Cortical plate
Primary somatosensory area mouth	SSp-m	Cortical plate
Primary somatosensory area nose	SSp-n	Cortical plate
Primary somatosensory area trunk	SSp-tr	Cortical plate
Primary somatosensory area upper limb	SSp-ul	Cortical plate
Primary visual area	VISp	Cortical plate
Retrosplenial area dorsal part	RSPd	Cortical plate
Retrosplenial area lateral agranular part	RSPagl	Cortical plate
Retrosplenial area ventral part	RSPv	Cortical plate
Secondary motor area	MOs	Cortical plate
Subiculum	SUB	Cortical plate
Supplemental somatosensory area	SSs	Cortical plate
Taenia tecta	TT	Cortical plate
Temporal association areas	TEa	Cortical plate
Ventral auditory area	AUDv	Cortical plate
Visceral area	VISC	Cortical plate
Basolateral amygdalar nucleus	BLA	Cortical subplate
Claustrum	CLA	Cortical subplate
Endopiriform nucleus	EP	Cortical subplate
Lateral amygdalar nucleus	LA	Cortical subplate
Posterior amygdalar nucleus	PA	Cortical subplate
Anterior amygdalar area	AAA	Striatum
Bed nucleus of the accessory olfactory tract	BA	Striatum
Caudoputamen	CP	Striatum
Central amygdalar nucleus	CEA	Striatum
Fundus of striatum	FS	Striatum
Intercalated amygdalar nucleus	IA	Striatum
Lateral septal complex	LSX	Striatum
Medial amygdalar nucleus	MEA	Striatum
Nucleus accumbens	ACB	Striatum
Olfactory tubercle	OT	Striatum
Septofimbrial nucleus	SF	Striatum
Bed nuclei of the stria terminalis	BST	Pallidum
Diagonal band nucleus	NDB	Pallidum
Globus pallidus external segment	GPe	Pallidum
Globus pallidus internal segment	GPi	Pallidum
Magnocellular nucleus	MA	Pallidum
Medial septal nucleus	MS	Pallidum
Substantia innominata	SI	Pallidum
Triangular nucleus of septum	TRS	Pallidum
Anterior group of the dorsal thalamus	ATN	Thalamus
Anterodorsal nucleus	AD	Thalamus
Anteroventral nucleus of thalamus	AV	Thalamus
Central lateral nucleus of the thalamus	CL	Thalamus
Central medial nucleus of the thalamus	CM	Thalamus
Dorsal part of the lateral geniculate complex	LGd	Thalamus
Interanterodorsal nucleus of the thalamus	IAD	Thalamus
Interanteromedial nucleus of the thalamus	IAM	Thalamus
Intergeniculate leaflet of the lateral geniculate complex	IGL	Thalamus
Intermediodorsal nucleus of the thalamus	IMD	Thalamus
Lateral dorsal nucleus of thalamus	LD	Thalamus
Lateral habenula	LH	Thalamus
Lateral posterior nucleus of the thalamus	LP	Thalamus
Medial geniculate complex	MG	Thalamus
Medial habenula	MH	Thalamus
Mediodorsal nucleus of thalamus	MD	Thalamus
Nucleus of reuniens	RE	Thalamus
Paracentral nucleus	PCN	Thalamus
Parafascicular nucleus	PF	Thalamus
Parataenial nucleus	PT	Thalamus
Paraventricular nucleus of the thalamus	PVT	Thalamus
Peripeduncular nucleus	PP	Thalamus
Posterior complex of the thalamus	PO	Thalamus
Posterior limiting nucleus of the thalamus	POL	Thalamus
Reticular nucleus of the thalamus	RT	Thalamus
Submedial nucleus of the thalamus	SMT	Thalamus
Subparafascicular nucleus	SPF	Thalamus
Thalamus sensory-motor cortex related	DORsm	Thalamus
Ventral anterior-lateral complex of the thalamus	VAL	Thalamus
Ventral medial nucleus of the thalamus	VM	Thalamus
Ventral part of the lateral geniculate complex	LGv	Thalamus
Ventral posterior complex of the thalamus	VP	Thalamus
Ventral posterolateral nucleus of the thalamus	VPL	Thalamus
Anterior hypothalamic nucleus	AHN	Hypothalamus
Anterodorsal preoptic nucleus	ADP	Hypothalamus
Anteroventral periventricular nucleus	AVPV	Hypothalamus
Anteroventral preoptic nucleus	AVP	Hypothalamus
Arcuate hypothalamic nucleus	ARH	Hypothalamus
Dorsal premammillary nucleus	PMd	Hypothalamus
Dorsomedial nucleus of the hypothalamus	DMH	Hypothalamus
Lateral hypothalamic area	LHA	Hypothalamus
Lateral preoptic area	LPO	Hypothalamus
Mammillary body	MBO	Hypothalamus
Medial preoptic area	MPO	Hypothalamus
Medial preoptic nucleus	MPN	Hypothalamus
Median preoptic nucleus	MEPO	Hypothalamus
Parastrial nucleus	PS	Hypothalamus
Parasubthalamic nucleus	PSTN	Hypothalamus
Paraventricular hypothalamic nucleus	PVH	Hypothalamus
Paraventricular hypothalamic nucleus descending division	PVHd	Hypothalamus
Periventricular hypothalamic nucleus posterior part	PVp	Hypothalamus
Periventricular hypothalamic nucleus preoptic part	PVpo	Hypothalamus
Periventricular zone	PVZ	Hypothalamus
Posterior hypothalamic nucleus	PH	Hypothalamus
Preparasubthalamic nucleus	PST	Hypothalamus
Retrochiasmatic area	RCH	Hypothalamus
Subparaventricular zone	SBPV	Hypothalamus
Subthalamic nucleus	STN	Hypothalamus
Suprachiasmatic nucleus	SCH	Hypothalamus
Supramammillary nucleus	SUM	Hypothalamus
Supraoptic nucleus	SO	Hypothalamus
Tuberal nucleus	TU	Hypothalamus
Ventrolateral preoptic nucleus	VLPO	Hypothalamus
Ventromedial hypothalamic nucleus	VMH	Hypothalamus
Zona incerta	ZI	Hypothalamus
Anterior pretectal nucleus	APN	Midbrain
Cuneiform nucleus	CUN	Midbrain
Inferior colliculus	IC	Midbrain
Interpeduncular nucleus	IPN	Midbrain
Medial pretectal area	MPT	Midbrain
Midbrain reticular nucleus	MRN	Midbrain
Midbrain reticular nucleus retrorubral area	RR	Midbrain
Nucleus of Darkschewitsch	ND	Midbrain
Nucleus of the brachium of the inferior colliculus	NB	Midbrain
Nucleus of the optic tract	NOT	Midbrain
Nucleus of the posterior commissure	NPC	Midbrain
Olivary pretectal nucleus	OP	Midbrain
Parabigeminal nucleus	PBG	Midbrain
Pedunculopontine nucleus	PPN	Midbrain
Periaqueductal gray	PAG	Midbrain
Posterior pretectal nucleus	PPT	Midbrain
Precommissural nucleus	PRC	Midbrain
Red nucleus	RN	Midbrain
Substantia nigra compact part	SNc	Midbrain
Substantia nigra reticular part	SNr	Midbrain
Superior colliculus motor related	SCm	Midbrain
Superior colliculus sensory related	SCs	Midbrain
Ventral tegmental area	VTA	Midbrain
Pons	P	Hindbrain
Pons motor related	P-mot	Hindbrain
Pontine reticular nucleus	PRNr	Hindbrain
Vestibular nuclei	VNC	Hindbrain
Ansiform lobule	AN	Cerebellum
Central lobule	CENT	Cerebellum
Culmen	CUL	Cerebellum
Paraflocculus	PFL	Cerebellum
Simple lobule	SIM	Cerebellum

#### Hierarchical clustering

Previous rat and mouse studies that examined functional connectivity used five to eight animals (Wheeler et al., 2013; Orsini et al., 2018). The number of samples that are examined in functional connectivity studies is the number of potential functional connections (i.e., 178 total brain regions all connecting with each other for each treatment). Furthermore, hierarchical clustering organizes brain regions into modules by grouping regions that show a similar functional connectivity profile across all other brain regions. Thus, more total functional connections minimize the effect that an inaccurate brain region-to-brain region functional connection has on network organization and overall network structure.

**Figure 3.**
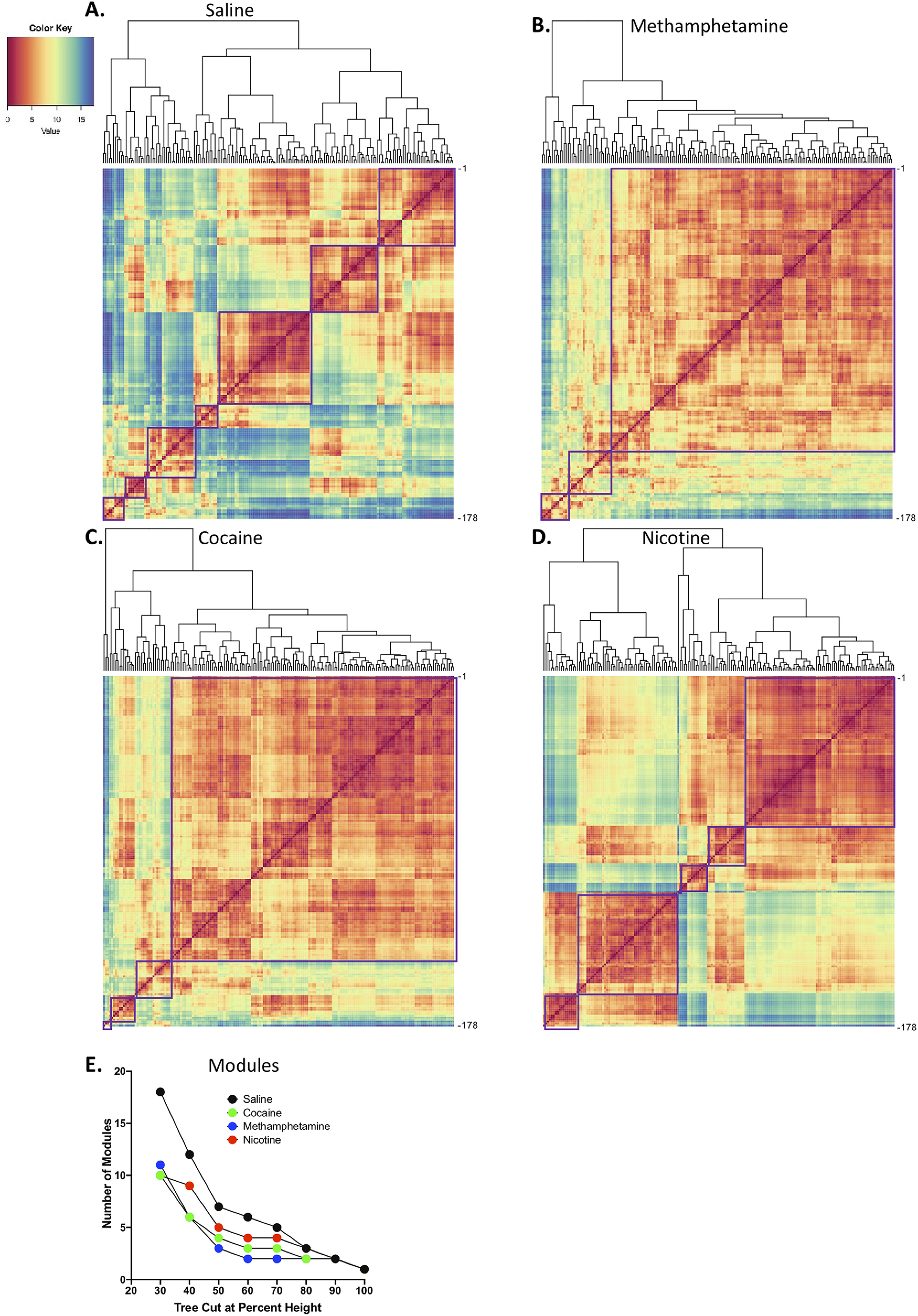
***A–D***, Hierarchical clustering of complete Euclidean distance matrices for each treatment. Modules were determined by cutting each dendrogram at half of the maximal tree height. ***A***, Relative distance of each brain region relative to the others that were examined in saline control mice. In control mice, seven distinct modules of coactivation were identified. ***B***, Relative distance of each brain region relative to the others that were examined in cocaine mice. In cocaine mice, four distinct modules of coactivation were identified. ***C***, Relative distance of each brain region relative to the others that were examined in methamphetamine mice. In methamphetamine mice, three distinct modules of coactivation were identified. ***D***, Relative distance of each brain region relative to the others that were examined in nicotine mice. In nicotine mice, five distinct modules of coactivation were identified. For all distance matrices, each module is boxed in purple. For the individual brain regions that are listed in panels ***A–D***, see Table 6. ***E***, Number of modules in each treatment condition after cutting the hierarchical clustered dendrogram at different percentages of tree height. In all cases (except at extreme cutoff values; e.g., 90–100%), the psychostimulant networks showed lower modularity compared with the control network. See Extended Data Figure 3-1 for correlation matrices for each treatment.

Interregional Pearson correlations were then used to calculate complete Euclidean distances between each pair of brain regions in each group of mice. The distance matrices were then hierarchically clustered using R Studio software by both row and column using the complete method to identify modules of functional connectivity within each treatment group. The hierarchical cluster dendrograms were trimmed at half the height of each given tree to split the dendrogram into specific modules. The result of a decrease in modularity that is attributable to psychostimulant use was consistent across multiple tree-cutting thresholds (Fig. 3*E*).

10.1523/ENEURO.0208-19.2021.f3-1 Extended Data Figure 3-1 Pearson correlation matrices for showing functional connectivity measures of each treatment. Download Figure 3-1, TIF file.

#### Graph theory identification of functional networks

We used a graph theory-based approach to identify the functional neural networks that were associated with each treatment condition. Graph theory is a branch of mathematics that is used to analyze complex networks, such as social, financial, protein, and neural networks (Jeong et al., 2001; Barabasi, 2009; Chiang et al., 2011; Varshney et al., 2011; Babu et al., 2012; Jarrell et al., 2012; Bargmann and Marder, 2013; Wheeler et al., 2013; Oh et al., 2014; Markov et al., 2014; Cohen and D’Esposito, 2016; Vetere et al., 2017). Using graph theory, functional networks can be delineated, and key brain regions of the network can be identified (Sporns et al., 2007; Rubinov and Sporns, 2010; Wheeler et al., 2013; Vetere et al., 2017).

Previous studies of regional functional connectivity profiles using Fos have focused on global measures of connectivity (e.g., degree; Wheeler et al., 2013). However, in correlation-based networks, these measures can be strongly influenced by the size of the subnetwork (module) in which a node participates (Power et al., 2013). For the graph theory analyses, we were interested in regional properties and not module size per se. Thus, module structure needs to be considered when examining the role that each region plays in the network. To accomplish this, we used two widely used centrality metrics that were designed for application to modular systems. The Z-scored version of within-module degree (WMDz) indexes the relative importance of a region within its own module (e.g., intramodule connectivity), and the participation coefficient (PC) indexes the extent to which a region connects diversely to multiple modules (e.g., intermodule connectivity; Guimera and Nunes Amaral, 2005).

We used the Pearson correlation values that were calculated for the brain regions from each treatment. Before plotting and calculating regional connectivity metrics, the network was thresholded to remove any edges that were weaker than *R *=* *0.75. As such, visualization and graph theory analyses were performed using only edges with positive weights. Regional connectivity metrics (PC and WMDz) were calculated as originally defined by Guimera and Nunes Amaral (2005), modified for application to networks with weighted edges. PC and WMDz were calculated using a customized version of the bctpy Python package (https://github.com/aestrivex/bctpy), which is derived from the MATLAB implementation of Brain Connectivity Toolbox (Rubinov and Sporns, 2010).

For WMDz, let $k_{i}$ (within-module degree) be the summed weight of all edges between region i and other regions in module $s_{i}$. Then, ${\bar{k}}_{s_{i}}$ is the average within-module degree of all regions in module $s_{i}$, and $\sigma_{k_{s_{i}}}$ is the standard deviation of those values. The WMDz is then defined as:

$$WMDz=\frac{k_{i}-{\bar{k}}_{s_{i}}}{\sigma_{k_{s_{i}}}}.$$

This provides a measure of the extent to which each region is connected to other regions in the same module.

For PC, let $k_{is}$ (between-module degree) be the summed weight of all edges between region i and regions in module s, and let $k_{i}$ (total degree) be the summed weight of all edges between region i and all other regions in the network. The PC of each region is then defined as:

$$P_{i}=1-\overset{N_{M}}{\underset{s=1}{\sum }}{\left(\frac{k_{is}}{k_{i}}\right)}^{2}.$$

This provides a measure of the extent to which the connections of a region are distributed mostly within its own module (PC approaching 0) or distributed evenly among all modules (PC approaching 1).

A high PC was considered ≥0.30, and a high WMDz was considered ≥0.80. Previous studies have used ranges of ≥0.30–0.80 for high PC and ≥1.5–2.5 for high WMDz (Guimera and Nunes Amaral, 2005; Cohen and D’Esposito, 2016). Because of differences in the sizes/types of networks that were examined and the methods that were used (e.g., Fos vs functional magnetic resonance imaging), we adjusted the range for consideration as having high PC and WMDz accordingly.

Network visualization was performed using a combination of Gephi 0.9.2 software (Bastian et al., 2009) and Adobe Illustrator software. Nodes were positioned using the Force Atlas 2 algorithm (Jacomy et al., 2014) with a handful of nodes that were repositioned manually for better visual organization.

## Results

### Psychostimulant withdrawal induces restructuring of brain functional networks

We examined the ways in which withdrawal from different psychostimulants alters functional connectivity and modular structuring of the brain. For an overview of the experimental design and analysis pipeline, see Figure 1. Representative examples Fos images collected can be seen in Figure 2. For all of the drugs tested, acute withdrawal produced widespread increases in the functional connectivity of brain regions compared with saline controls (Fig. 3*A–D*). Importantly, modular structuring of the brain decreased in response to withdrawal from each psychostimulant compared with controls. When using a threshold of 50% of tree height, saline control mice exhibited a modular structure of the brain that contained seven modules, whereas cocaine mice had four modules, methamphetamine mice had three modules, and nicotine mice had five modules and one isolated brain region that was not grouped with any other region (i.e., interanterodorsal nucleus of the thalamus; Fig. 3*A–E*). Notably, the decrease in the number of modules during withdrawal was independent of the clustering thresholds that were used (Fig. 3*E*). These data indicate that psychostimulant withdrawal decreases modularity of the functional network compared with controls.

### Characterization of individual network features

To further characterize the features of each individual network, we used a graph theory approach to identify potential hub brain regions with the most intramodular and intermodular connectivity, which may drive activity within the network and thus be critical for neuronal function in the withdrawal state. We examined positive connectivity (thresholded to a Pearson correlation coefficient >0.75 [0.75R] for inclusion as a network connection) for the network for each treatment and used the modular organization that was identified by hierarchical clustering to partition the regions of the networks. The 0.75R threshold was chosen because all of the brain regions in each network showed connections to other regions at this threshold. Previous animal model studies used various thresholds, ranging from 0.3R to 0.85R (Wheeler et al., 2013; Orsini et al., 2018), to examine connectivity. Negative network connectivity was not examined herein because the precise meaning of such connectivity is controversial and thus is not often examined in network-based approaches (Giove et al., 2009; Meunier et al., 2009; Murphy et al., 2009; Chen et al., 2011).

**Figure 4.**
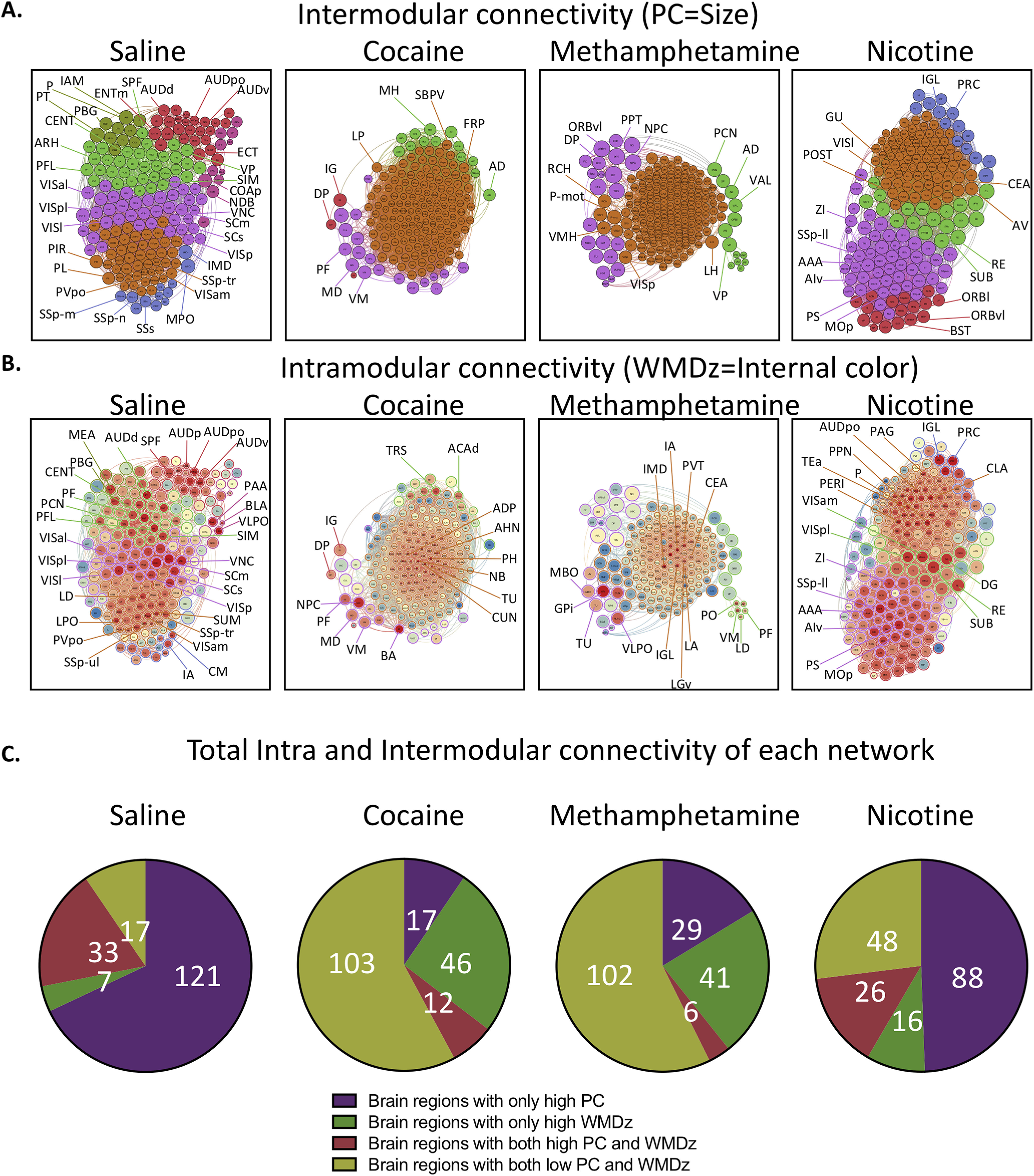
Intramodular (WMDz) and intermodular (PC) network features of each treatment. A high PC was considered ≥0.30, and a high WMDz was considered ≥0.80. ***A***, Highlights of several regions with high PC in each module of each network (see Table 1 for names of abbreviations). ***B***, Highlights of several regions with high WMDz (red, higher; blue, lower) in each module of each network. Note that the WMDz color intensity is only relative to the other regions within the same network and not other networks (see Table 1 for names of abbreviations). ***C***, Total number of brain regions that accounted for high PC, high WMDz, or both in each network. The control and nicotine networks showed much greater intermodular connectivity and a greater number of regions with both high intermodular and intramodular connectivity. The cocaine and methamphetamine networks showed higher levels of intramodular connectivity and a low number of regions with intermodular connectivity.

**Figure 5.**
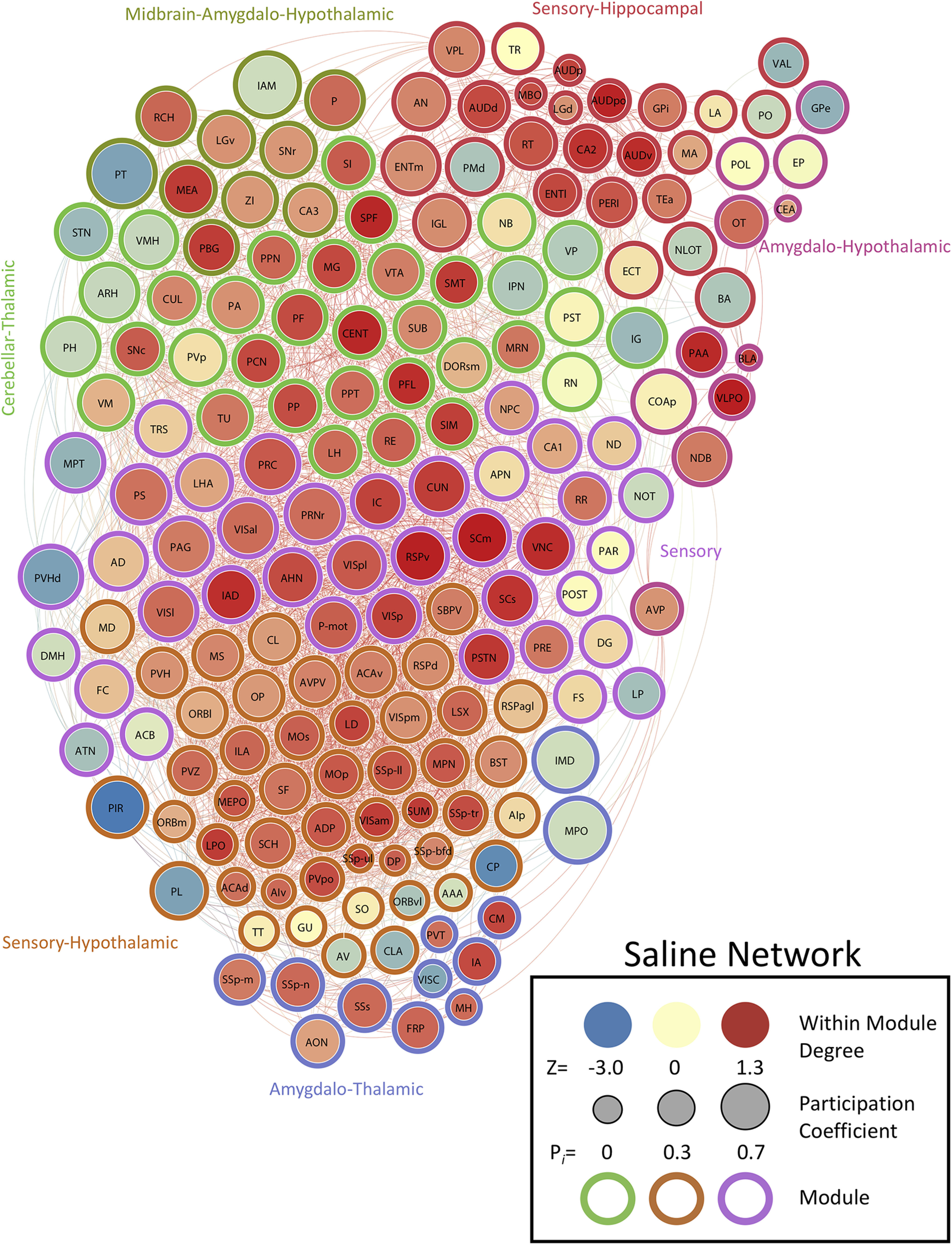
Neural network of control mice thresholded to 0.75R. Nodes/brain regions of the network are represented by circles. The size of the node represents the PC (smaller, lower PC; larger, higher PC). The internal color of each circle represents the WMDz (dark blue, lowest; dark red, highest). The color of the modules that are identified in Figure 1*C* are represented by different colored edges. See figure key for examples of each representative component of the figure.

**Figure 6.**
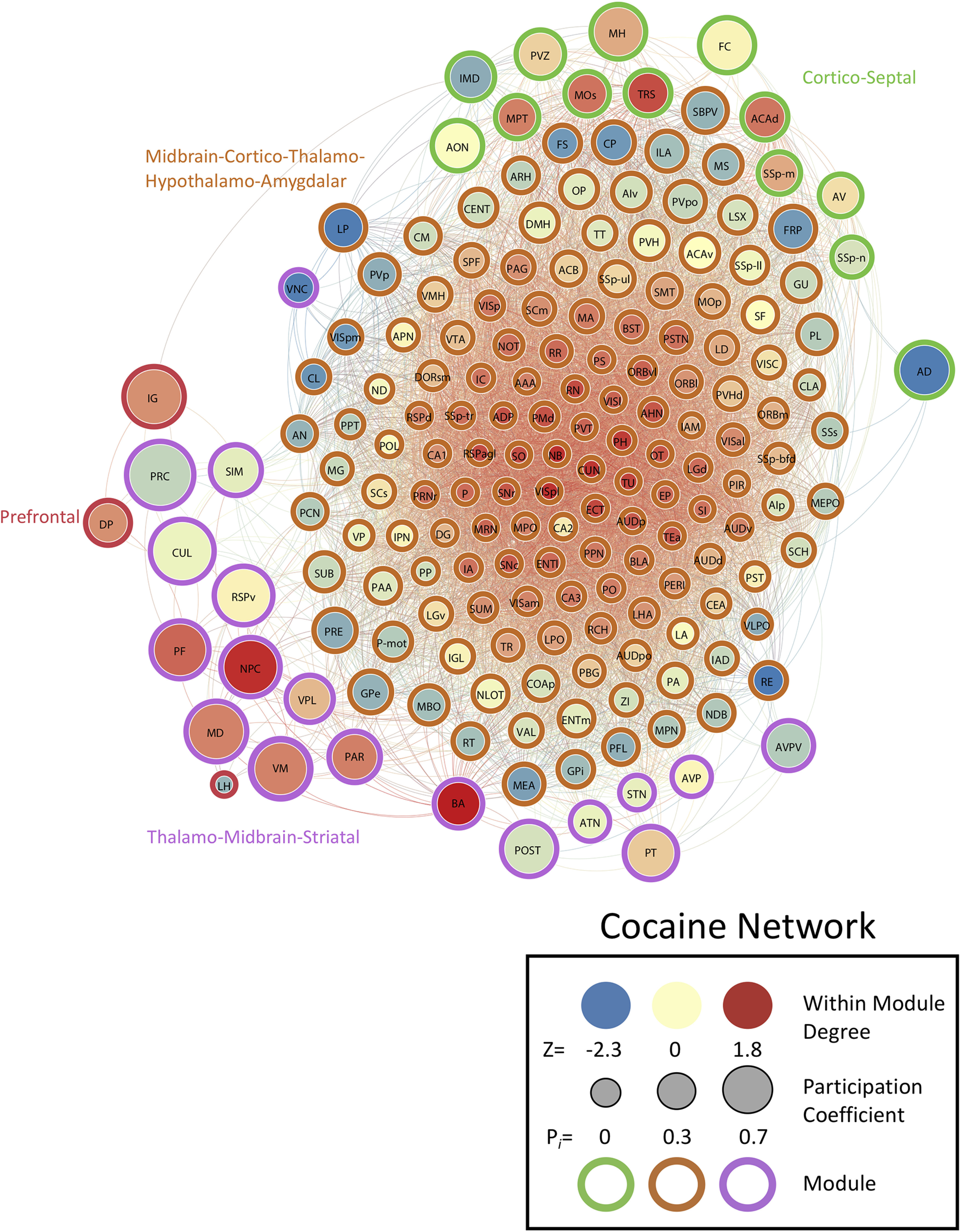
Neural network of cocaine mice during withdrawal thresholded to 0.75R. Nodes/brain regions of the network are represented by circles. The size of the node represents the PC (smaller, lower PC; larger, higher PC). The internal color of each circle represents the WMDz (dark blue, lowest; dark red, highest). The color of the modules that are identified in Figure 1*D* are represented by different colored edges. See figure key for examples of each representative component of the figure.

**Figure 7.**
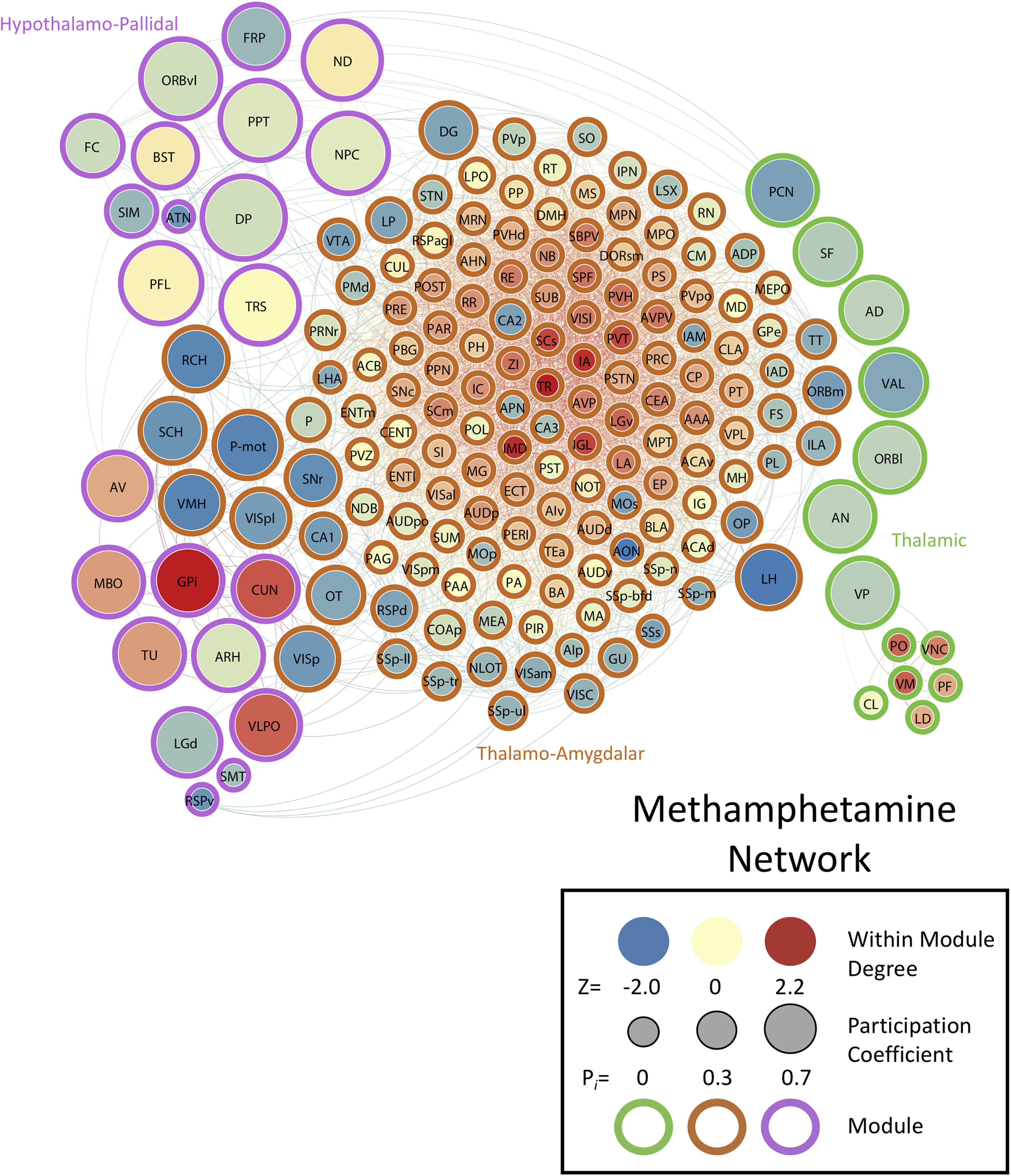
Neural network of methamphetamine mice during withdrawal thresholded to 0.75R. Nodes/brain regions of the network are represented by circles. The size of the node represents the PC (smaller, lower PC; larger, higher PC). The internal color of each circle represents the WMDz (dark blue, lowest; dark red, highest). The color of the modules that are identified in Figure 1*E* are represented by different colored edges. See figure key for examples of each representative component of the figure.

**Figure 8.**
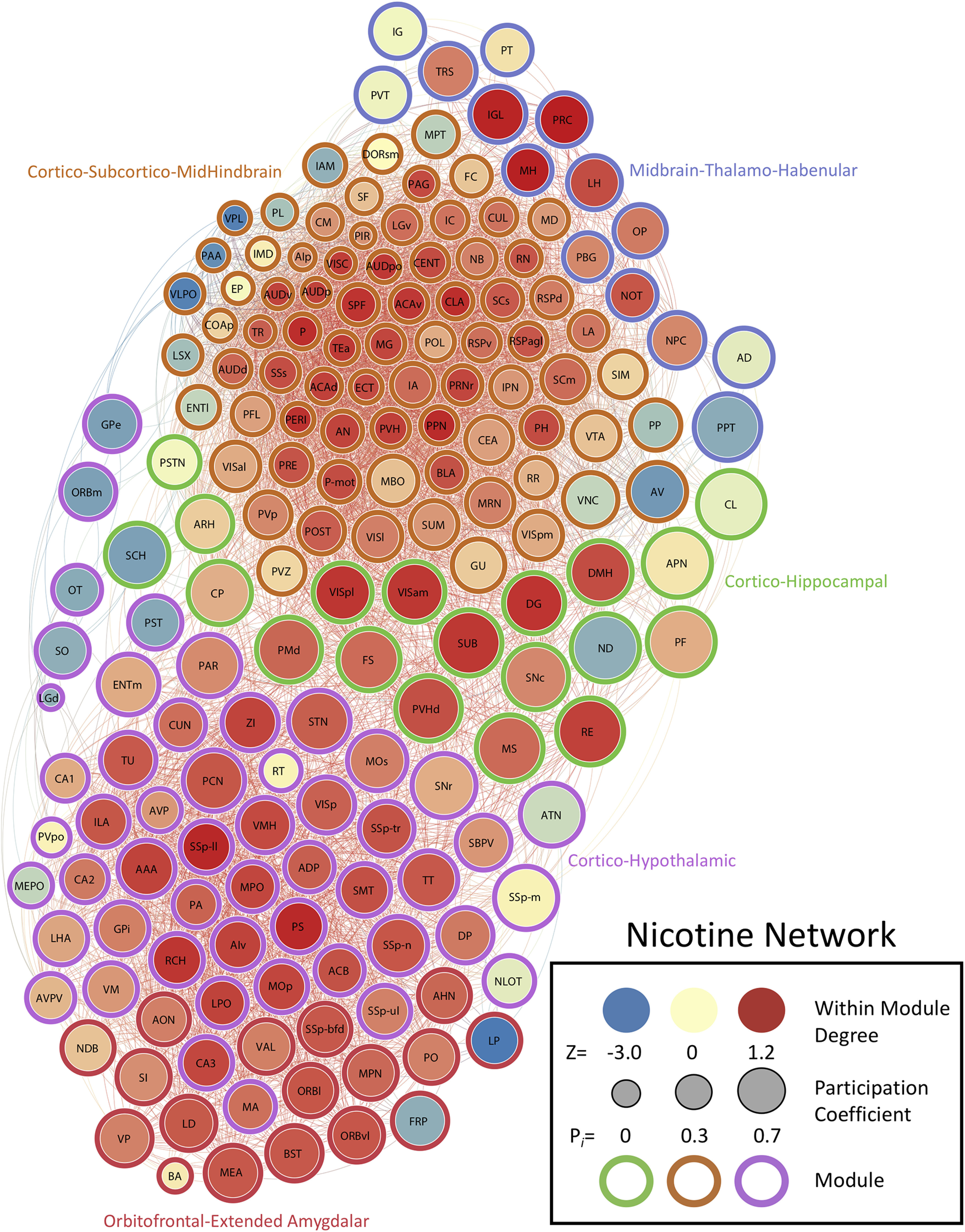
Neural network of nicotine mice during withdrawal thresholded to 0.75R. Nodes/brain regions of the network are represented by circles. The size of the node represents the PC (smaller, lower PC; larger, higher PC). The internal color of each circle represents the WMDz (dark blue, lowest; dark red, highest). The color of the modules that are identified in Figure 1*F* are represented by different colored edges. See figure key for examples of each representative component of the figure.

We determined the PC (i.e., a measure of importance for intermodular connectivity) and the WMDz (i.e., a measure of importance for intramodular connectivity; Guimera and Nunes Amaral, 2005) for all brain regions in the networks. A high PC was considered ≥0.30, and a high WMDz was considered ≥0.80. Overall, the control and nicotine networks showed much greater intermodular connectivity (high PC) and a great number of regions with both high intermodular and intramodular connectivity (high PC and WMDz). The cocaine and methamphetamine networks showed higher levels of intramodular connectivity (high WMDz) and a low number of regions with intermodular connectivity (Fig. 4*A-C*). We named each module in each network based on the group of brain regions with the highest WMDz score in the module and considered these regions to be drivers of activity within individual modules (Figs. 5-8 for names).

### The control network is driven by sensory-motor regions

The saline control network had 3176 total functional connections and consisted of seven modules, many of which were heavily driven by sensory-motor brain regions. Of these seven modules, five contained several sensory or motor brain regions that were ranked in the top five for intramodular connectivity (high WMDz). In most cases, a separate set of thalamic brain regions was responsible for intermodular connectivity (high PC; see Table 2 for a full list of values for the network). Overall, the control network had more brain regions with high WMDz, high PC, or both in individual modules compared with other networks. This indicates a more interconnected network with more hub regions (Figs. 2, 3).

**Table 2:** Saline network values

Brain region	Module	PC	WMDz
Agranular insular area posterior part	3	0.35	−0.48
Agranular insular area ventral part	3	0.15	0.56
Anterior cingulate area dorsal part	3	0.22	0.49
Anterior cingulate area ventral part	3	0.47	0.34
Anterior olfactory nucleus	4	0.50	0.02
Anterolateral visual area	1	0.65	0.53
Anteromedial visual area	3	0.31	0.99
Cortical amygdalar area posterior part	7	0.72	−0.69
Dentate gyrus	1	0.49	−0.46
Dorsal auditory area	5	0.48	0.87
Dorsal peduncular area	3	0.08	0.66
Ectorhinal area	5	0.58	−0.57
Entorhinal area lateral part	5	0.40	0.89
Entorhinal area medial part	5	0.63	0.20
Fasciola cinerea	1	0.60	−0.26
Field CA1	1	0.52	−0.04
Field CA2	5	0.41	1.11
Field CA3	6	0.52	0.08
Frontal pole cerebral cortex	4	0.46	0.59
Gustatory areas	3	0.26	−0.79
Induseum griseum	2	0.64	−1.94
Infralimbic area	3	0.42	0.59
Lateral visual area	1	0.63	0.54
Nucleus of the lateral olfactory tract	5	0.50	−1.57
Orbital area lateral part	3	0.47	−0.09
Orbital area medial part	3	0.29	−0.10
Orbital area ventrolateral part	3	0.25	−1.76
Parasubiculum	1	0.38	−0.78
Perirhinal area	5	0.44	0.75
Piriform area	3	0.66	−2.98
Piriform-amygdalar area	7	0.44	1.21
Posterior auditory area	5	0.32	1.25
Posterolateral visual area	1	0.61	0.72
Posteromedial visual area	3	0.46	0.19
Postpiriform transition area	5	0.47	−0.83
Postsubiculum	1	0.34	−0.78
Prelimbic area	3	0.59	−2.32
Presubiculum	1	0.49	0.42
Primary auditory area	5	0.09	0.94
Primary motor area	3	0.42	0.73
Primary somatosensory area barrel field	3	0.19	0.28
Primary somatosensory area lower limb	3	0.41	0.76
Primary somatosensory area mouth	4	0.45	0.40
Primary somatosensory area nose	4	0.50	0.57
Primary somatosensory area trunk	3	0.34	0.84
Primary somatosensory area upper limb	3	0.04	0.97
Primary visual area	1	0.53	0.94
Retrosplenial area dorsal part	3	0.47	0.22
Retrosplenial area lateral agranular part	3	0.49	−0.27
Retrosplenial area ventral part	1	0.59	1.30
Secondary motor area	3	0.42	0.65
Subiculum	2	0.50	0.25
Supplemental somatosensory area	4	0.50	0.56
Taenia tecta	3	0.25	−0.68
Temporal association areas	5	0.40	0.38
Ventral auditory area	5	0.32	1.17
Visceral area	4	0.21	−2.23
Basolateral amygdalar nucleus	7	0.00	0.99
Claustrum	3	0.39	−1.94
Endopiriform nucleus	7	0.50	−0.93
Lateral amygdalar nucleus	5	0.28	−0.60
Posterior amygdalar nucleus	2	0.57	0.18
Anterior amygdalar area	3	0.21	−1.32
Bed nucleus of the accessory olfactory tract	5	0.62	−1.74
Caudoputamen	3	0.46	−2.69
Central amygdalar nucleus	7	0.00	−0.01
Fundus of striatum	1	0.49	−0.47
Intercalated amygdalar nucleus	4	0.39	0.87
Lateral septal complex	3	0.43	0.60
Medial amygdalar nucleus	6	0.58	1.02
Nucleus accumbens	1	0.48	−1.18
Olfactory tubercle	7	0.50	0.51
Septofimbrial nucleus	3	0.46	0.44
Bed nuclei of the stria terminalis	3	0.46	0.21
Diagonal band nucleus	7	0.62	0.39
Globus pallidus external segment	7	0.48	−2.08
Globus pallidus internal segment	5	0.39	0.38
Magnocellular nucleus	5	0.31	−0.02
Medial septal nucleus	3	0.47	0.29
Substantia innominata	2	0.50	0.69
Triangular nucleus of septum	1	0.58	−0.37
Anterior group of the dorsal thalamus	1	0.50	−1.82
Anterodorsal nucleus	1	0.62	−0.25
Anteroventral nucleus of thalamus	3	0.33	−1.54
Central lateral nucleus of the thalamus	3	0.48	0.09
Central medial nucleus of the thalamus	4	0.29	0.91
Dorsal part of the lateral geniculate complex	5	0.13	0.17
Interanterodorsal nucleus of the thalamus	1	0.59	1.15
Interanteromedial nucleus of the thalamus	6	0.77	−1.38
Intergeniculate leaflet of the lateral geniculate complex	5	0.60	0.22
Intermediodorsal nucleus of the thalamus	4	0.75	−1.36
Lateral dorsal nucleus of thalamus	3	0.34	0.94
Lateral habenula	2	0.50	0.53
Lateral posterior nucleus of the thalamus	1	0.47	−1.86
Medial geniculate complex	2	0.50	0.88
Medial habenula	4	0.18	0.55
Mediodorsal nucleus of thalamus	3	0.51	−0.33
Nucleus of reuniens	2	0.49	0.64
Paracentral nucleus	2	0.49	0.88
Parafascicular nucleus	2	0.55	0.85
Parataenial nucleus	6	0.82	−2.30
Paraventricular nucleus of the thalamus	4	0.18	0.49
Peripeduncular nucleus	2	0.50	0.83
Posterior complex of the thalamus	5	0.40	−1.44
Posterior limiting nucleus of the thalamus	7	0.48	−0.86
Reticular nucleus of the thalamus	5	0.50	0.77
Submedial nucleus of the thalamus	2	0.47	1.02
Subparafascicular nucleus	2	0.48	1.22
Thalamus sensory-motor cortex related	2	0.53	−0.14
Ventral anterior-lateral complex of the thalamus	5	0.43	−1.98
Ventral medial nucleus of the thalamus	2	0.57	−0.13
Ventral part of the lateral geniculate complex	6	0.59	0.18
Ventral posterior complex of the thalamus	2	0.61	−1.67
Ventral posterolateral nucleus of the thalamus	5	0.54	0.28
Anterior hypothalamic nucleus	1	0.61	0.85
Anterodorsal preoptic nucleus	3	0.41	0.72
Anteroventral periventricular nucleus	3	0.46	0.32
Anteroventral preoptic nucleus	7	0.49	0.16
Arcuate hypothalamic nucleus	2	0.63	−1.47
Dorsal premammillary nucleus	5	0.60	−1.76
Dorsomedial nucleus of the hypothalamus	1	0.49	−1.35
Lateral hypothalamic area	1	0.59	−0.01
Lateral preoptic area	3	0.28	1.02
Mammillary body	5	0.10	0.84
Medial preoptic area	4	0.77	−1.38
Medial preoptic nucleus	3	0.41	0.72
Median preoptic nucleus	3	0.26	0.76
Parastrial nucleus	1	0.65	0.47
Parasubthalamic nucleus	1	0.53	1.06
Paraventricular hypothalamic nucleus	3	0.46	0.18
Paraventricular hypothalamic nucleus descending division	1	0.72	−2.34
Periventricular hypothalamic nucleus posterior part	2	0.58	−0.54
Periventricular hypothalamic nucleus preoptic part	3	0.33	0.84
Periventricular zone	3	0.43	0.53
Posterior hypothalamic nucleus	2	0.63	−1.46
Preparasubthalamic nucleus	2	0.59	−0.72
Retrochiasmatic area	6	0.63	0.59
Subparaventricular zone	3	0.46	0.36
Subthalamic nucleus	2	0.58	−1.93
Suprachiasmatic nucleus	3	0.44	0.53
Supramammillary nucleus	3	0.16	1.01
Supraoptic nucleus	3	0.28	−0.67
Tuberal nucleus	2	0.56	0.42
Ventrolateral preoptic nucleus	7	0.36	1.31
Ventromedial hypothalamic nucleus	2	0.61	−1.47
Zona incerta	6	0.56	0.11
Anterior pretectal nucleus	1	0.52	−0.52
Cuneiform nucleus	1	0.63	0.99
Inferior colliculus	1	0.53	0.95
Interpeduncular nucleus	2	0.61	−1.72
Medial pretectal area	1	0.63	−2.05
Midbrain reticular nucleus	2	0.48	0.43
Midbrain reticular nucleus retrorubral area	1	0.50	0.44
Nucleus of Darkschewitsch	1	0.49	−0.33
Nucleus of the brachium of the inferior colliculus	2	0.59	−0.55
Nucleus of the optic tract	1	0.52	−1.41
Nucleus of the posterior commissure	1	0.50	0.04
Olivary pretectal nucleus	3	0.47	0.12
Parabigeminal nucleus	6	0.54	1.00
Pedunculopontine nucleus	2	0.49	0.60
Periaqueductal gray	1	0.64	0.39
Posterior pretectal nucleus	2	0.50	0.52
Precommissural nucleus	1	0.64	0.72
Red nucleus	2	0.60	−0.92
Substantia nigra compact part	2	0.50	0.69
Substantia nigra reticular part	6	0.57	0.13
Superior colliculus motor related	1	0.60	1.32
Superior colliculus sensory related	1	0.54	1.10
Ventral tegmental area	2	0.56	0.37
Pons	6	0.63	0.57
Pons motor related	1	0.55	0.67
Pontine reticular nucleus	1	0.65	0.52
Vestibular nuclei	1	0.58	1.16
Ansiform lobule	5	0.60	0.30
Central lobule	2	0.51	1.22
Culmen	2	0.51	0.28
Paraflocculus	2	0.46	1.16
Simple lobule	2	0.47	0.98

### The cocaine withdrawal network is driven by cortico-thalamo-hypothalamic regions

The cocaine network had 7127 total functional connections and consisted of four modules, one with the majority of all brain regions and three others with a small subset of regions. In the large module (module 1; 144 brain regions), nearly one-third (32%) of the total brain regions within the module (i.e., a mixed set of midbrain-cortico-thalamic-hypothalamic-amygdalar brain regions) had high WMDz. The brain regions that drive intramodular connectivity (high WMDz) in this module did not have any intermodular connectivity (PC). Interestingly, only three brain regions in this module (subparaventricular zone, lateral posterior nucleus of the thalamus, and frontal pole cerebral cortex) reached the criterion (PC ≥ 0.30) for a high level of intermodular connectivity, suggesting sparse communication with other modules.

One of the smaller modules, a septal (triangular nucleus of the septum) and cortical (e.g., secondary motor area and dorsal anterior cingulate area) module (module 3) had a different set of thalamic brain regions that had high PC. The other two smaller modules, a prefrontal-habenular module [module 4; dorsal peduncular area (DP), induseum griseum, and lateral habenula] and a thalamic (parafascicular nucleus, mediodorsal nucleus of the thalamus, and ventral medial nucleus of the thalamus), midbrain (nucleus of the posterior commissure), and striatal (bed nucleus of the accessory olfactory tract) module (module 2) contained regions with both a high WMDz and high PC, suggesting that these regions may be potential hubs within the network. Overall, the cocaine network contained the highest number of functional connections in any network but had minimal interconnection between modules (Figs. 2, 4; see Table 3 for a full list of values for the network).

**Table 3:** Cocaine network values

Brain region	Module	PC	WMDz
Agranular insular area posterior part	1	0.07	−0.69
Agranular insular area ventral part	1	0.22	−0.76
Anterior cingulate area dorsal part	3	0.34	0.93
Anterior cingulate area ventral part	1	0.21	−0.30
Anterior olfactory nucleus	3	0.41	−0.32
Anterolateral visual area	1	0.09	0.74
Anteromedial visual area	1	0.05	0.80
Cortical amygdalar area posterior part	1	0.16	−0.72
Dentate gyrus	1	0.02	0.36
Dorsal auditory area	1	0.05	0.35
Dorsal peduncular area	4	0.32	0.71
Ectorhinal area	1	0.01	1.37
Entorhinal area lateral part	1	0.04	1.05
Entorhinal area medial part	1	0.17	−0.58
Fasciola cinerea	3	0.47	−0.19
Field CA1	1	0.02	0.71
Field CA2	1	0.02	−0.11
Field CA3	1	0.05	0.89
Frontal pole cerebral cortex	1	0.32	−1.86
Gustatory areas	1	0.21	−0.85
Induseum griseum	4	0.55	0.71
Infralimbic area	1	0.25	−1.28
Lateral visual area	1	0.00	1.11
Nucleus of the lateral olfactory tract	1	0.18	−0.41
Orbital area lateral part	1	0.10	0.66
Orbital area medial part	1	0.07	0.39
Orbital area ventrolateral part	1	0.04	1.10
Parasubiculum	2	0.53	−0.16
Perirhinal area	1	0.05	0.61
Piriform area	1	0.00	0.47
Piriform-amygdalar area	1	0.17	−0.63
Posterior auditory area	1	0.07	0.11
Posterolateral visual area	1	0.00	1.58
Posteromedial visual area	1	0.13	−1.88
Postpiriform transition area	1	0.10	0.52
Postsubiculum	2	0.60	−0.47
Prelimbic area	1	0.23	−1.08
Presubiculum	1	0.28	−1.55
Primary auditory area	1	0.00	1.19
Primary motor area	1	0.12	0.37
Primary somatosensory area barrel field	1	0.07	0.31
Primary somatosensory area lower limb	1	0.19	−0.44
Primary somatosensory area mouth	3	0.29	0.47
Primary somatosensory area nose	3	0.25	−0.72
Primary somatosensory area trunk	1	0.02	1.01
Primary somatosensory area upper limb	1	0.16	0.07
Primary visual area	1	0.06	0.92
Retrosplenial area dorsal part	1	0.02	0.80
Retrosplenial area lateral agranular part	1	0.00	1.32
Retrosplenial area ventral part	2	0.17	−0.53
Secondary motor area	3	0.32	0.95
Subiculum	1	0.22	−0.86
Supplemental somatosensory area	1	0.14	−1.08
Taenia tecta	1	0.13	−0.63
Temporal association areas	1	0.01	1.19
Ventral auditory area	1	0.02	0.81
Visceral area	1	0.12	−0.03
Basolateral amygdalar nucleus	1	0.03	0.83
Claustrum	1	0.08	−0.82
Endopiriform nucleus	1	0.00	1.06
Lateral amygdalar nucleus	1	0.07	−0.35
Posterior amygdalar nucleus	1	0.14	−0.55
Anterior amygdalar area	1	0.00	0.83
Bed nucleus of the accessory olfactory tract	2	0.42	0.84
Caudoputamen	1	0.27	−1.88
Central amygdalar nucleus	1	0.06	0.13
Fundus of striatum	1	0.19	−1.88
Intercalated amygdalar nucleus	1	0.03	0.97
Lateral septal complex	1	0.20	−0.72
Medial amygdalar nucleus	1	0.24	−1.74
Nucleus accumbens	1	0.15	0.13
Olfactory tubercle	1	0.00	1.23
Septofimbrial nucleus	1	0.17	−0.31
Bed nuclei of the stria terminalis	1	0.11	0.91
Diagonal band nucleus	1	0.17	−1.14
Globus pallidus external segment	1	0.28	−1.46
Globus pallidus internal segment	1	0.18	−1.30
Magnocellular nucleus	1	0.12	0.87
Medial septal nucleus	1	0.21	−1.35
Substantia innominata	1	0.00	0.95
Triangular nucleus of septum	3	0.34	1.31
Anterior group of the dorsal thalamus	2	0.38	1.78
Anterodorsal nucleus	3	0.48	−2.30
Anteroventral nucleus of thalamus	3	0.29	0.00
Central lateral nucleus of the thalamus	1	0.13	−1.96
Central medial nucleus of the thalamus	1	0.16	−0.82
Dorsal part of the lateral geniculate complex	1	0.03	1.01
Interanterodorsal nucleus of the thalamus	1	0.11	−0.89
Interanteromedial nucleus of the thalamus	1	0.00	0.42
Intergeniculate leaflet of the lateral geniculate complex	1	0.12	−0.18
Intermediodorsal nucleus of the thalamus	3	0.36	−1.56
Lateral dorsal nucleus of thalamus	1	0.12	0.47
Lateral habenula	4	0.00	−1.41
Lateral posterior nucleus of the thalamus	1	0.32	−2.30
Medial geniculate complex	1	0.10	−0.86
Medial habenula	3	0.47	0.46
Mediodorsal nucleus of thalamus	2	0.55	0.82
Nucleus of reuniens	1	0.19	−2.35
Paracentral nucleus	1	0.16	−1.09
Parafascicular nucleus	2	0.45	0.18
Parataenial nucleus	2	0.24	−0.20
Paraventricular nucleus of the thalamus	1	0.00	1.09
Peripeduncular nucleus	1	0.03	−0.79
Posterior complex of the thalamus	1	0.03	0.85
Posterior limiting nucleus of the thalamus	1	0.02	−0.23
Reticular nucleus of the thalamus	1	0.17	−1.27
Submedial nucleus of the thalamus	1	0.15	0.50
Subparafascicular nucleus	1	0.12	0.33
Thalamus sensory-motor cortex related	1	0.07	0.30
Ventral anterior-lateral complex of the thalamus	1	0.13	−0.70
Ventral medial nucleus of the thalamus	2	0.66	−0.94
Ventral part of the lateral geniculate complex	1	0.11	−0.02
Ventral posterior complex of the thalamus	1	0.07	−0.50
Ventral posterolateral nucleus of the thalamus	2	0.40	−1.10
Anterior hypothalamic nucleus	1	0.06	1.22
Anterodorsal preoptic nucleus	1	0.00	1.28
Anteroventral periventricular nucleus	2	0.54	1.62
Anteroventral preoptic nucleus	2	0.52	1.08
Arcuate hypothalamic nucleus	1	0.17	−0.98
Dorsal premammillary nucleus	1	0.06	1.26
Dorsomedial nucleus of the hypothalamus	1	0.21	−0.49
Lateral hypothalamic area	1	0.07	0.67
Lateral preoptic area	1	0.10	0.53
Mammillary body	1	0.22	−1.28
Medial preoptic area	1	0.02	0.83
Medial preoptic nucleus	1	0.15	−1.12
Median preoptic nucleus	1	0.13	−1.07
Parastrial nucleus	1	0.02	0.96
Parasubthalamic nucleus	1	0.10	0.97
Paraventricular hypothalamic nucleus	1	0.22	−0.40
Paraventricular hypothalamic nucleus descending division	1	0.13	0.22
Periventricular hypothalamic nucleus posterior part	1	0.24	−1.54
Periventricular hypothalamic nucleus preoptic part	1	0.25	−0.91
Periventricular zone	3	0.38	0.12
Posterior hypothalamic nucleus	1	0.04	1.47
Preparasubthalamic nucleus	1	0.06	−0.14
Retrochiasmatic area	1	0.06	0.70
Subparaventricular zone	1	0.30	−1.46
Subthalamic nucleus	2	0.48	−0.73
Suprachiasmatic nucleus	1	0.12	−0.77
Supramammillary nucleus	1	0.06	0.69
Supraoptic nucleus	1	0.00	1.32
Tuberal nucleus	1	0.00	1.40
Ventrolateral preoptic nucleus	1	0.07	−1.56
Ventromedial hypothalamic nucleus	1	0.13	0.16
Zona incerta	1	0.11	−0.75
Anterior pretectal nucleus	1	0.10	−0.21
Cuneiform nucleus	1	0.00	1.54
Inferior colliculus	1	0.03	0.94
Interpeduncular nucleus	1	0.06	−0.20
Medial pretectal area	3	0.30	0.84
Midbrain reticular nucleus	1	0.00	1.08
Midbrain reticular nucleus retrorubral area	1	0.09	1.03
Nucleus of Darkschewitsch	1	0.05	−0.40
Nucleus of the brachium of the inferior colliculus	1	0.00	1.60
Nucleus of the optic tract	1	0.07	0.98
Nucleus of the posterior commissure	2	0.36	0.33
Olivary pretectal nucleus	1	0.21	−0.61
Parabigeminal nucleus	1	0.09	0.20
Pedunculopontine nucleus	1	0.05	0.90
Periaqueductal gray	1	0.12	0.70
Posterior pretectal nucleus	1	0.06	−1.07
Precommissural nucleus	2	0.24	−0.50
Red nucleus	1	0.01	1.32
Substantia nigra compact part	1	0.01	1.18
Substantia nigra reticular part	1	0.00	1.23
Superior colliculus motor related	1	0.11	0.71
Superior colliculus sensory related	1	0.11	−0.05
Ventral tegmental area	1	0.11	0.26
Pons	1	0.01	1.03
Pons motor related	1	0.21	−1.09
Pontine reticular nucleus	1	0.02	0.98
Vestibular nuclei	2	0.19	−2.26
Ansiform lobule	1	0.15	−1.44
Central lobule	1	0.20	−0.79
Culmen	2	0.56	0.82
Paraflocculus	1	0.17	−1.50
Simple lobule	2	0.41	−0.57

### The methamphetamine withdrawal network is driven by thalamic regions

The methamphetamine network had 3182 functional connections and consisted of three modules, one with the majority of all brain regions and two others with a small subset of regions. In the large module (module 1), a group of thalamic (e.g., intermediodorsal nucleus of the thalamus, paraventricular nucleus of the thalamus, intergeniculate leaflet of the lateral geniculate complex, and ventral part of the lateral geniculate complex) and amygdalar (intercalated amygdala, central amygdala, and lateral amygdala) regions had high WMDz, but these brain regions did not have any intermodular connectivity (PC), and a separate set of hypothalamic, cortical, and mid/hindbrain regions was responsible for intermodular connectivity.

The second module (module 2) had several hypothalamic (e.g., mammillary body, ventrolateral preoptic nucleus, and tuberal nucleus) and pallidal (globus pallidus and internal segment) brain regions with high WMDz and a separate set of cortical regions (e.g., DP and orbital area, ventral part) and midbrain regions (e.g., posterior pretectal nucleus, nucleus of the posterior commissure, and nucleus of Darkschewitsch) that had high interconnectivity with other modules (high PC).

The third module (module 3), a thalamic module, had several thalamic regions with high WMDz (e.g., ventral medial nucleus of the thalamus, posterior complex of the thalamus, parafascicular nucleus, and lateral dorsal nucleus of the thalamus). Interestingly, within this module, a separate set of thalamic regions (e.g., paracentral nucleus, ventral anterior-lateral complex of the thalamus, ventral posterior complex of the thalamus, and anterodorsal nucleus) had high PC, indicating that this module is internally directed by thalamic regions and also externally communicates through these regions. Overall, the methamphetamine network had a similar number of total connections to the control network, but it had minimal interconnections between modules (Figs. 2, 5; see Table 4 for a full list of values for the network).

**Table 4:** Methamphetamine network values

Brain region	Module	PC	WMDz
Agranular insular area posterior part	1	0.00	−0.90
Agranular insular area ventral part	1	0.00	0.64
Anterior cingulate area dorsal part	1	0.00	−0.08
Anterior cingulate area ventral part	1	0.00	0.41
Anterior olfactory nucleus	1	0.00	−2.01
Anterolateral visual area	1	0.00	0.65
Anteromedial visual area	1	0.08	−1.15
Cortical amygdalar area posterior part	1	0.09	−0.41
Dentate gyrus	1	0.32	−1.31
Dorsal auditory area	1	0.00	0.67
Dorsal peduncular area	2	0.67	−0.41
Ectorhinal area	1	0.00	0.81
Entorhinal area lateral part	1	0.00	0.70
Entorhinal area medial part	1	0.00	−0.26
Fasciola cinerea	2	0.38	−0.46
Field CA1	1	0.18	−1.45
Field CA2	1	0.10	−1.36
Field CA3	1	0.00	−0.84
Frontal pole cerebral cortex	2	0.43	−1.01
Gustatory areas	1	0.07	−1.09
Induseum griseum	1	0.00	0.05
Infralimbic area	1	0.13	−1.04
Lateral visual area	1	0.00	1.30
Nucleus of the lateral olfactory tract	1	0.07	−1.03
Orbital area lateral part	3	0.50	−0.59
Orbital area medial part	1	0.18	−1.44
Orbital area ventrolateral part	2	0.63	−0.41
Parasubiculum	1	0.00	0.95
Perirhinal area	1	0.00	0.61
Piriform area	1	0.00	−0.10
Piriform-amygdalar area	1	0.00	0.11
Posterior auditory area	1	0.05	−0.25
Posterolateral visual area	1	0.35	−1.44
Posteromedial visual area	1	0.00	−0.29
Postpiriform transition area	1	0.00	2.19
Postsubiculum	1	0.00	0.92
Prelimbic area	1	0.00	−1.01
Presubiculum	1	0.00	0.98
Primary auditory area	1	0.00	1.01
Primary motor area	1	0.00	−0.94
Primary somatosensory area barrel field	1	0.00	−0.18
Primary somatosensory area lower limb	1	0.08	−1.08
Primary somatosensory area mouth	1	0.00	−1.12
Primary somatosensory area nose	1	0.00	−0.26
Primary somatosensory area trunk	1	0.07	−0.97
Primary somatosensory area upper limb	1	0.07	−1.08
Primary visual area	1	0.41	−1.52
Retrosplenial area dorsal part	1	0.17	−1.33
Retrosplenial area lateral agranular part	1	0.04	0.18
Retrosplenial area ventral part	2	0.00	−1.56
Secondary motor area	1	0.00	−1.40
Subiculum	1	0.00	1.00
Supplemental somatosensory area	1	0.00	−1.37
Taenia tecta	1	0.09	−1.18
Temporal association areas	1	0.00	0.66
Ventral auditory area	1	0.00	0.15
Visceral area	1	0.07	−1.11
Basolateral amygdalar nucleus	1	0.00	0.35
Claustrum	1	0.00	0.54
Endopiriform nucleus	1	0.00	0.93
Lateral amygdalar nucleus	1	0.00	1.26
Posterior amygdalar nucleus	1	0.00	0.01
Anterior amygdalar area	1	0.00	1.07
Bed nucleus of the accessory olfactory tract	1	0.00	0.51
Caudoputamen	1	0.00	0.87
Central amygdalar nucleus	1	0.00	1.21
Fundus of striatum	1	0.06	−0.84
Intercalated amygdalar nucleus	1	0.00	2.04
Lateral septal complex	1	0.06	−0.83
Medial amygdalar nucleus	1	0.06	−0.61
Nucleus accumbens	1	0.00	−0.05
Olfactory tubercle	1	0.32	−1.28
Septofimbrial nucleus	3	0.45	−0.71
Bed nuclei of the stria terminalis	2	0.43	0.28
Diagonal band nucleus	1	0.05	−0.24
Globus pallidus external segment	1	0.00	−0.20
Globus pallidus internal segment	2	0.50	2.18
Magnocellular nucleus	1	0.00	−0.13
Medial septal nucleus	1	0.00	0.49
Substantia innominata	1	0.00	0.66
Triangular nucleus of septum	2	0.61	0.15
Anterior group of the dorsal thalamus	2	0.00	−1.56
Anterodorsal nucleus	3	0.45	−0.61
Anteroventral nucleus of thalamus	2	0.48	0.85
Central lateral nucleus of the thalamus	3	0.00	−0.03
Central medial nucleus of the thalamus	1	0.00	−0.30
Dorsal part of the lateral geniculate complex	2	0.47	−0.90
Interanterodorsal nucleus of the thalamus	1	0.00	−0.67
Interanteromedial nucleus of the thalamus	1	0.00	−1.28
Intergeniculate leaflet of the lateral geniculate complex	1	0.00	1.80
Intermediodorsal nucleus of the thalamus	1	0.00	2.04
Lateral dorsal nucleus of thalamus	3	0.00	0.81
Lateral habenula	1	0.41	−1.97
Lateral posterior nucleus of the thalamus	1	0.16	−1.32
Medial geniculate complex	1	0.00	0.87
Medial habenula	1	0.00	−0.19
Mediodorsal nucleus of thalamus	1	0.00	0.08
Nucleus of reuniens	1	0.00	1.27
Paracentral nucleus	3	0.50	−1.38
Parafascicular nucleus	3	0.00	0.85
Parataenial nucleus	1	0.00	0.74
Paraventricular nucleus of the thalamus	1	0.00	1.82
Peripeduncular nucleus	1	0.00	0.33
Posterior complex of the thalamus	3	0.00	1.48
Posterior limiting nucleus of the thalamus	1	0.00	−0.12
Reticular nucleus of the thalamus	1	0.04	−0.11
Submedial nucleus of the thalamus	2	0.00	−0.90
Subparafascicular nucleus	1	0.00	1.36
Thalamus sensory-motor cortex related	1	0.00	0.57
Ventral anterior-lateral complex of the thalamus	3	0.45	−1.28
Ventral medial nucleus of the thalamus	3	0.00	1.54
Ventral part of the lateral geniculate complex	1	0.00	1.48
Ventral posterior complex of the thalamus	3	0.50	−0.68
Ventral posterolateral nucleus of the thalamus	1	0.00	0.54
Anterior hypothalamic nucleus	1	0.00	0.69
Anterodorsal preoptic nucleus	1	0.06	−0.84
Anteroventral periventricular nucleus	1	0.00	1.34
Anteroventral preoptic nucleus	1	0.00	1.23
Arcuate hypothalamic nucleus	2	0.50	−0.32
Dorsal premammillary nucleus	1	0.07	−0.82
Dorsomedial nucleus of the hypothalamus	1	0.00	0.39
Lateral hypothalamic area	1	0.00	−1.22
Lateral preoptic area	1	0.04	0.14
Mammillary body	2	0.50	0.98
Medial preoptic area	1	0.00	0.53
Medial preoptic nucleus	1	0.00	0.75
Median preoptic nucleus	1	0.00	−0.14
Parastrial nucleus	1	0.00	0.86
Parasubthalamic nucleus	1	0.00	0.82
Paraventricular hypothalamic nucleus	1	0.00	1.36
Paraventricular hypothalamic nucleus descending division	1	0.00	0.75
Periventricular hypothalamic nucleus posterior part	1	0.11	−0.62
Periventricular hypothalamic nucleus preoptic part	1	0.00	0.65
Periventricular zone	1	0.00	0.14
Posterior hypothalamic nucleus	1	0.00	0.52
Preparasubthalamic nucleus	1	0.00	−0.08
Retrochiasmatic area	1	0.43	−1.79
Subparaventricular zone	1	0.00	1.26
Subthalamic nucleus	1	0.07	−0.83
Suprachiasmatic nucleus	1	0.44	−1.61
Supramammillary nucleus	1	0.00	−0.11
Supraoptic nucleus	1	0.07	−0.73
Tuberal nucleus	2	0.50	0.97
Ventrolateral preoptic nucleus	2	0.49	1.53
Ventromedial hypothalamic nucleus	1	0.41	−1.83
Zona incerta	1	0.00	1.35
Anterior pretectal nucleus	1	0.00	−0.98
Cuneiform nucleus	2	0.44	1.64
Inferior colliculus	1	0.00	1.08
Interpeduncular nucleus	1	0.05	−0.36
Medial pretectal area	1	0.00	0.45
Midbrain reticular nucleus	1	0.00	0.65
Midbrain reticular nucleus retrorubral area	1	0.00	1.08
Nucleus of Darkschewitsch	2	0.60	0.27
Nucleus of the brachium of the inferior colliculus	1	0.00	1.09
Nucleus of the optic tract	1	0.00	0.27
Nucleus of the posterior commissure	2	0.63	−0.23
Olivary pretectal nucleus	1	0.10	−1.47
Parabigeminal nucleus	1	0.00	0.57
Pedunculopontine nucleus	1	0.00	0.86
Periaqueductal gray	1	0.00	−0.01
Posterior pretectal nucleus	2	0.63	−0.27
Precommissural nucleus	1	0.00	0.85
Red nucleus	1	0.04	−0.18
Substantia nigra compact part	1	0.00	0.80
Substantia nigra reticular part	1	0.30	−1.69
Superior colliculus motor related	1	0.00	1.18
Superior colliculus sensory related	1	0.00	1.79
Ventral tegmental area	1	0.11	−1.43
Pons	1	0.14	−0.54
Pons motor related	1	0.47	−1.84
Pontine reticular nucleus	1	0.09	−0.34
Vestibular nuclei	3	0.00	1.21
Ansiform lobule	3	0.50	−0.60
Central lobule	1	0.00	0.14
Culmen	1	0.00	0.23
Paraflocculus	2	0.64	0.22
Simple lobule	2	0.26	−1.03

### The nicotine withdrawal network is driven by cortical and extended amygdalar regions

The nicotine network had 4957 functional connections, the second most of all conditions, and consisted of five modules and one brain region (interanterodorsal nucleus of the thalamus) that was disconnected from the entire network. Overall, the nicotine network was relatively interconnected between modules and had two large modules and three medium modules.

One of the large modules (module 1) contained midbrain (e.g., pedunculopontine nucleus and periaqueductal gray), hindbrain (e.g., pons and pontine reticular nucleus), cortical (e.g., perirhinal area, posterior auditory area, ventral anterior cingulate temporal association areas, and visceral area), and subcortical (claustrum) brain regions that had high WMDz. A separate set of cortical (e.g., postsubiculum, lateral visual area, and gustatory areas), thalamic (e.g., anteroventral nucleus of the thalamus and peripeduncular nucleus), hypothalamic (e.g., posterior periventricular nucleus, supramammillary nucleus, and periventricular zone), and midbrain (e.g., midbrain reticular nucleus, ventral tegmental area, and medial pretectal area) brain regions and a few others that included the central amygdala and vestibular nuclei had high PC.

In the second large module (module 4), a set of sensory/cortical [e.g., primary somatosensory area, lower limb, ventral agranular insular area (AIv), and primary motor area] and hypothalamic (e.g., parastriatal nucleus, retrochiasmatic area, lateral preoptic area, medial preoptic area, and zona incerta) brain regions had high WMDz. All of the same sensory/cortical and hypothalamic regions had high PC and a number of other thalamic and sensory regions. Additionally, the anterior amygdalar area (AAA) also showed both high WMDz and high PC.

One of the smaller modules (module 2) consisted of hippocampal (dentate gyrus) and sensory/cortical (e.g., posterolateral visual area, anteromedial visual area, and subiculum [SUB]) regions, along with the nucleus of reuniens (RE) with high WMDz. The SUB and RE also had high PC, along with other thalamic, hypothalamic, and midbrain regions.

In another smaller module (module 3), the precommissural nucleus (PRC), medial habenula, and intergeniculate leaflet of the lateral geniculate complex (IGL) had high WMDz and high PC. Other midbrain and thalamic regions also had high PC.

In the last small module (module 5), no regions reached the criterion for high WMDz, but the orbitofrontal cortex (lateral and ventrolateral orbital area), bed nucleus of the stria terminalis, and medial amygdalar nucleus were all in the top five values (WMDz = 0.64–0.67). However, every region in this module, with the exception of the bed nucleus of the accessory olfactory tract, reached the criterion for high PC (Figs. 2, 6; see Table 5 for a full list of values for the network).

**Table 5:** Nicotine network values

Region	Module	PC	WMDz
Agranular insular area posterior part	1	0.03	0.12
Agranular insular area ventral part	4	0.43	0.85
Anterior cingulate area dorsal part	1	0.17	0.87
Anterior cingulate area ventral part	1	0.22	0.94
Anterior olfactory nucleus	5	0.42	0.27
Anterolateral visual area	1	0.38	−0.12
Anteromedial visual area	2	0.57	0.97
Cortical amygdalar area posterior part	1	0.12	−0.46
Dentate gyrus	2	0.58	0.99
Dorsal auditory area	1	0.20	0.52
Dorsal peduncular area	4	0.49	0.33
Ectorhinal area	1	0.13	0.78
Entorhinal area lateral part	1	0.28	−1.50
Entorhinal area medial part	4	0.57	−0.12
Fasciola cinerea	1	0.25	−0.35
Field CA1	4	0.34	−0.11
Field CA2	4	0.36	0.33
Field CA3	4	0.44	0.80
Frontal pole cerebral cortex	5	0.49	−2.12
Gustatory areas	1	0.44	−0.40
Induseum griseum	3	0.47	−1.01
Infralimbic area	4	0.42	0.66
Lateral visual area	1	0.38	0.27
Nucleus of the lateral olfactory tract	4	0.41	−1.16
Orbital area lateral part	5	0.44	0.67
Orbital area medial part	4	0.48	−2.31
Orbital area ventrolateral part	5	0.46	0.66
Parasubiculum	4	0.59	0.18
Perirhinal area	1	0.13	1.02
Piriform area	1	0.00	0.11
Piriform-amygdalar area	1	0.11	−2.62
Posterior auditory area	1	0.12	0.95
Posterolateral visual area	2	0.55	0.98
Posteromedial visual area	1	0.38	−0.12
Postpiriform transition area	1	0.10	0.58
Postsubiculum	1	0.34	0.58
Prelimbic area	1	0.16	−1.81
Presubiculum	1	0.27	0.69
Primary auditory area	1	0.07	0.85
Primary motor area	4	0.43	0.82
Primary somatosensory area barrel field	5	0.43	0.58
Primary somatosensory area lower limb	4	0.44	1.12
Primary somatosensory area mouth	4	0.61	−0.75
Primary somatosensory area nose	4	0.54	0.67
Primary somatosensory area trunk	4	0.48	0.70
Primary somatosensory area upper limb	4	0.44	0.26
Primary visual area	4	0.50	0.56
Retrosplenial area dorsal part	1	0.26	0.30
Retrosplenial area lateral agranular part	1	0.21	0.67
Retrosplenial area ventral part	1	0.20	0.41
Secondary motor area	4	0.55	0.27
Subiculum	2	0.66	0.97
Supplemental somatosensory area	1	0.22	0.73
Taenia tecta	4	0.53	0.65
Temporal association areas	1	0.14	0.93
Ventral auditory area	1	0.09	0.85
Visceral area	1	0.09	0.90
Basolateral amygdalar nucleus	1	0.25	0.71
Claustrum	1	0.18	1.06
Endopiriform nucleus	1	0.10	−0.96
Lateral amygdalar nucleus	1	0.24	0.36
Posterior amygdalar nucleus	4	0.35	0.54
Anterior amygdalar area	4	0.52	0.86
Bed nucleus of the accessory olfactory tract	5	0.13	−0.68
Caudoputamen	2	0.61	−0.17
Central amygdalar nucleus	1	0.36	−0.01
Fundus of striatum	2	0.59	0.47
Intercalated amygdalar nucleus	1	0.29	0.46
Lateral septal complex	1	0.22	−1.88
Medial amygdalar nucleus	5	0.49	0.64
Nucleus accumbens	4	0.39	0.70
Olfactory tubercle	4	0.39	−2.16
Septofimbrial nucleus	1	0.16	−0.31
Bed nuclei of the stria terminalis	5	0.49	0.65
Diagonal band nucleus	5	0.44	−0.33
Globus pallidus external segment	4	0.49	−2.31
Globus pallidus internal segment	4	0.45	0.27
Magnocellular nucleus	4	0.41	0.43
Medial septal nucleus	2	0.66	0.48
Substantia innominata	5	0.46	0.14
Triangular nucleus of septum	3	0.49	0.28
Anterior group of the dorsal thalamus	4	0.60	−1.42
Anterodorsal nucleus	3	0.53	−1.16
Anteroventral nucleus of thalamus	1	0.53	−2.45
Central lateral nucleus of the thalamus	2	0.67	−1.12
Central medial nucleus of the thalamus	1	0.17	0.09
Dorsal part of the lateral geniculate complex	4	0.00	−2.07
Interanteromedial nucleus of the thalamus	1	0.27	−2.07
Intergeniculate leaflet of the lateral geniculate complex	3	0.50	1.16
Intermediodorsal nucleus of the thalamus	1	0.12	−0.71
Lateral dorsal nucleus of thalamus	5	0.47	0.64
Lateral habenula	3	0.47	0.75
Lateral posterior nucleus of the thalamus	5	0.44	−2.94
Medial geniculate complex	1	0.22	0.81
Medial habenula	3	0.38	1.19
Mediodorsal nucleus of thalamus	1	0.23	0.03
Nucleus of reuniens	2	0.67	0.86
Paracentral nucleus	4	0.58	0.66
Parafascicular nucleus	2	0.68	−0.14
Parataenial nucleus	3	0.40	−0.61
Paraventricular nucleus of the thalamus	3	0.46	−1.06
Peripeduncular nucleus	1	0.41	−1.81
Posterior complex of the thalamus	5	0.45	0.26
Posterior limiting nucleus of the thalamus	1	0.21	−0.13
Reticular nucleus of the thalamus	4	0.25	−0.76
Submedial nucleus of the thalamus	4	0.45	0.72
Subparafascicular nucleus	1	0.25	0.99
Thalamus sensory-motor cortex related	1	0.20	−0.83
Ventral anterior-lateral complex of the thalamus	5	0.46	0.27
Ventral medial nucleus of the thalamus	4	0.45	0.06
Ventral part of the lateral geniculate complex	1	0.21	0.44
Ventral posterior complex of the thalamus	5	0.48	0.26
Ventral posterolateral nucleus of the thalamus	1	0.12	−2.84
Anterior hypothalamic nucleus	5	0.44	0.51
Anterodorsal preoptic nucleus	4	0.40	0.58
Anteroventral periventricular nucleus	4	0.38	−0.22
Anteroventral preoptic nucleus	4	0.30	0.05
Arcuate hypothalamic nucleus	2	0.49	−0.42
Dorsal premammillary nucleus	2	0.64	0.50
Dorsomedial nucleus of the hypothalamus	2	0.61	0.75
Lateral hypothalamic area	4	0.44	−0.06
Lateral preoptic area	4	0.40	0.85
Mammillary body	1	0.39	−0.30
Medial preoptic area	4	0.40	0.81
Medial preoptic nucleus	5	0.45	0.50
Median preoptic nucleus	4	0.29	−1.51
Parastrial nucleus	4	0.45	1.11
Parasubthalamic nucleus	2	0.42	−0.99
Paraventricular hypothalamic nucleus	1	0.22	0.88
Paraventricular hypothalamic nucleus descending division	2	0.65	0.72
Periventricular hypothalamic nucleus posterior part	1	0.34	0.21
Periventricular hypothalamic nucleus preoptic part	4	0.21	−0.74
Periventricular zone	1	0.35	−0.50
Posterior hypothalamic nucleus	1	0.28	0.76
Preparasubthalamic nucleus	4	0.45	−2.20
Retrochiasmatic area	4	0.45	0.98
Subparaventricular zone	4	0.48	0.07
Subthalamic nucleus	4	0.59	0.58
Suprachiasmatic nucleus	2	0.61	−2.32
Supramammillary nucleus	1	0.37	0.05
Supraoptic nucleus	4	0.45	−2.10
Tuberal nucleus	4	0.49	0.64
Ventrolateral preoptic nucleus	1	0.22	−2.82
Ventromedial hypothalamic nucleus	4	0.49	0.77
Zona incerta	4	0.50	0.85
Anterior pretectal nucleus	2	0.68	−0.63
Cuneiform nucleus	4	0.40	0.44
Inferior colliculus	1	0.19	0.38
Interpeduncular nucleus	1	0.26	0.06
Medial pretectal area	1	0.33	−1.59
Midbrain reticular nucleus	1	0.37	0.26
Midbrain reticular nucleus retrorubral area	1	0.26	−0.19
Nucleus of Darkschewitsch	2	0.69	−2.09
Nucleus of the brachium of the inferior colliculus	1	0.22	0.26
Nucleus of the optic tract	3	0.40	0.67
Nucleus of the posterior commissure	3	0.49	0.20
Olivary pretectal nucleus	3	0.44	0.33
Parabigeminal nucleus	3	0.38	0.20
Pedunculopontine nucleus	1	0.21	1.08
Periaqueductal gray	1	0.15	0.78
Posterior pretectal nucleus	3	0.66	−2.16
Precommissural nucleus	3	0.46	1.21
Red nucleus	1	0.17	0.68
Substantia nigra compact part	2	0.64	0.19
Substantia nigra reticular part	4	0.62	−0.15
Superior colliculus motor related	1	0.36	0.45
Superior colliculus sensory related	1	0.25	0.64
Ventral tegmental area	1	0.37	−0.33
Pons	1	0.18	1.06
Pons motor related	1	0.26	0.75
Pontine reticular nucleus	1	0.23	0.82
Vestibular nuclei	1	0.47	−1.51
Ansiform lobule	1	0.25	0.84
Central lobule	1	0.20	0.76
Culmen	1	0.19	0.40
Paraflocculus	1	0.27	−0.03
Simple lobule	1	0.36	−0.48

**Table 6:** Top to bottom order of brain regions in **Figure 1**

Number	Saline hierarchical order	Cocaine hierarchical order
1	Retrosplenial area ventral part	Inferior colliculus
2	Interanterodorsal nucleus of the thalamus	Primary visual area
3	Anterior hypothalamic nucleus	Nucleus of the optic tract
4	Posterolateral visual area	Thalamus sensory-motor cortex related
5	Precommissural nucleus	Retrosplenial area dorsal part
6	Superior colliculus motor related	Field CA1
7	Cuneiform nucleus	Retrosplenial area lateral agranular part
8	Primary visual area	Anterior amygdalar area
9	Superior colliculus sensory related	Anterodorsal preoptic nucleus
10	Parasubthalamic nucleus	Primary somatosensory area trunk
11	Vestibular nuclei	Interanteromedial nucleus of the thalamus
12	Pons motor related	Subparafascicular nucleus
13	Lateral visual area	Superior colliculus motor related
14	Anterolateral visual area	Periaqueductal gray
15	Pontine reticular nucleus	Magnocellular nucleus
16	Periaqueductal gray	Bed nuclei of the stria terminalis
17	Parastrial nucleus	Midbrain reticular nucleus retrorubral area
18	Fasciola cinerea	Ventromedial hypothalamic nucleus
19	Anterodorsal nucleus	Ventral tegmental area
20	Triangular nucleus of septum	Anterior pretectal nucleus
21	Lateral hypothalamic area	Endopiriform nucleus
22	Dorsomedial nucleus of the hypothalamus	Olfactory tubercle
23	Nucleus accumbens	Tuberal nucleus
24	Anterior group of the dorsal thalamus	Piriform area
25	Paraventricular hypothalamic nucleus descending division	Substantia innominata
26	Medial pretectal area	Ventral auditory area
27	Postsubiculum	Dorsal part of the lateral geniculate complex
28	Parasubiculum	Posterolateral visual area
29	Nucleus of the optic tract	Nucleus of the brachium of the inferior colliculus
30	Midbrain reticular nucleus retrorubral area	Supraoptic nucleus
31	Inferior colliculus	Cuneiform nucleus
32	Anterior pretectal nucleus	Paraventricular nucleus of the thalamus
33	Nucleus of Darkschewitsch	Lateral visual area
34	Field CA1	Orbital area ventrolateral part
35	Nucleus of the posterior commissure	Red nucleus
36	Fundus of striatum	Parastrial nucleus
37	Dentate gyrus	Parasubthalamic nucleus
38	Presubiculum	Anterior hypothalamic nucleus
39	Lateral posterior nucleus of the thalamus	Posterior hypothalamic nucleus
40	Parafascicular nucleus	Dorsal premammillary nucleus
41	Peripeduncular nucleus	Lateral hypothalamic area
42	Central lobule	Retrochiasmatic area
43	Posterior pretectal nucleus	Perirhinal area
44	Lateral habenula	Field CA3
45	Nucleus of reuniens	Posterior complex of the thalamus
46	Ventral medial nucleus of the thalamus	Entorhinal area lateral part
47	Tuberal nucleus	Intercalated amygdalar nucleus
48	Periventricular hypothalamic nucleus posterior part	Substantia nigra compact part
49	Posterior amygdalar nucleus	Basolateral amygdalar nucleus
50	Ventromedial hypothalamic nucleus	Pedunculopontine nucleus
51	Posterior hypothalamic nucleus	Medial preoptic area
52	Arcuate hypothalamic nucleus	Ectorhinal area
53	Subthalamic nucleus	Primary auditory area
54	Paracentral nucleus	Temporal association areas
55	Substantia nigra compact part	Pontine reticular nucleus
56	Culmen	Substantia nigra reticular part
57	Pedunculopontine nucleus	Pons
58	Interpeduncular nucleus	Midbrain reticular nucleus
59	Ventral posterior complex of the thalamus	Field CA2
60	Induseum griseum	Supramammillary nucleus
61	Preparasubthalamic nucleus	Anteromedial visual area
62	Nucleus of the brachium of the inferior colliculus	Posterior auditory area
63	Red nucleus	Visceral area
64	Ventral tegmental area	Primary motor area
65	Substantia innominata	Paraventricular hypothalamic nucleus descending division
66	Medial geniculate complex	Lateral dorsal nucleus of thalamus
67	Subiculum	Primary somatosensory area barrel field
68	Midbrain reticular nucleus	Orbital area medial part
69	Thalamus sensory-motor cortex related	Orbital area lateral part
70	Simple lobule	Anterolateral visual area
71	Paraflocculus	Median preoptic nucleus
72	Submedial nucleus of the thalamus	Suprachiasmatic nucleus
73	Subparafascicular nucleus	Supplemental somatosensory area
74	Olivary pretectal nucleus	Agranular insular area posterior part
75	Central lateral nucleus of the thalamus	Primary somatosensory area lower limb
76	Medial septal nucleus	Septofimbrial nucleus
77	Subparaventricular zone	Anterior cingulate area ventral part
78	Anterior cingulate area ventral part	Paraventricular hypothalamic nucleus
79	Secondary motor area	Primary somatosensory area upper limb
80	Suprachiasmatic nucleus	Submedial nucleus of the thalamus
81	Periventricular zone	Nucleus accumbens
82	Septofimbrial nucleus	Claustrum
83	Paraventricular hypothalamic nucleus	Agranular insular area ventral part
84	Orbital area lateral part	Lateral septal complex
85	Mediodorsal nucleus of thalamus	Taenia tecta
86	Posteromedial visual area	Arcuate hypothalamic nucleus
87	Retrosplenial area dorsal part	Olivary pretectal nucleus
88	Anteroventral periventricular nucleus	Dorsomedial nucleus of the hypothalamus
89	Bed nuclei of the stria terminalis	Prelimbic area
90	Retrosplenial area lateral agranular part	Periventricular hypothalamic nucleus preoptic part
91	Medial preoptic nucleus	Gustatory areas
92	Anterodorsal preoptic nucleus	Frontal pole cerebral cortex
93	Primary motor area	Subparaventricular zone
94	Lateral septal complex	Caudoputamen
95	Primary somatosensory area lower limb	Fundus of striatum
96	Lateral dorsal nucleus of thalamus	Infralimbic area
97	Primary somatosensory area trunk	Medial septal nucleus
98	Anteromedial visual area	Central lateral nucleus of the thalamus
99	Lateral preoptic area	Posteromedial visual area
100	Periventricular hypothalamic nucleus preoptic part	Lateral posterior nucleus of the thalamus
101	Median preoptic nucleus	Central lobule
102	Infralimbic area	Central medial nucleus of the thalamus
103	Primary somatosensory area upper limb	Periventricular hypothalamic nucleus posterior part
104	Supramammillary nucleus	Cortical amygdalar area posterior part
105	Gustatory areas	Nucleus of the lateral olfactory tract
106	Taenia tecta	Entorhinal area medial part
107	Supraoptic nucleus	Zona incerta
108	Claustrum	Ventral anterior-lateral complex of the thalamus
109	Anteroventral nucleus of thalamus	Posterior amygdalar nucleus
110	Prelimbic area	Postpiriform transition area
111	Piriform area	Lateral preoptic area
112	Agranular insular area ventral part	Parabigeminal nucleus
113	Dorsal peduncular area	Intergeniculate leaflet of the lateral geniculate complex
114	Anterior cingulate area dorsal part	Ventral part of the lateral geniculate complex
115	Orbital area medial part	Interanterodorsal nucleus of the thalamus
116	Orbital area ventrolateral part	Lateral amygdalar nucleus
117	Anterior amygdalar area	Ventrolateral preoptic nucleus
118	Caudoputamen	Central amygdalar nucleus
119	Primary somatosensory area barrel field	Dorsal auditory area
120	Agranular insular area posterior part	Preparasubthalamic nucleus
121	Paraventricular nucleus of the thalamus	Ventral posterior complex of the thalamus
122	Medial habenula	Interpeduncular nucleus
123	Frontal pole cerebral cortex	Peripeduncular nucleus
124	Anterior olfactory nucleus	Dentate gyrus
125	Central medial nucleus of the thalamus	Superior colliculus sensory related
126	Intercalated amygdalar nucleus	Piriform-amygdalar area
127	Medial preoptic area	Medial geniculate complex
128	Intermediodorsal nucleus of the thalamus	Posterior pretectal nucleus
129	Supplemental somatosensory area	Nucleus of Darkschewitsch
130	Primary somatosensory area nose	Posterior limiting nucleus of the thalamus
131	Primary somatosensory area mouth	Paracentral nucleus
132	Visceral area	Subiculum
133	Dorsal auditory area	Ansiform lobule
134	Entorhinal area lateral part	Diagonal band nucleus
135	Field CA2	Medial preoptic nucleus
136	Mammillary body	Paraflocculus
137	Posterior auditory area	Medial amygdalar nucleus
138	Ventral auditory area	Globus pallidus internal segment
139	Temporal association areas	Nucleus of reuniens
140	Ventral posterolateral nucleus of the thalamus	Mammillary body
141	Ansiform lobule	Globus pallidus external segment
142	Entorhinal area medial part	Reticular nucleus of the thalamus
143	Intergeniculate leaflet of the lateral geniculate complex	Presubiculum
144	Perirhinal area	Pons motor related
145	Reticular nucleus of the thalamus	Mediodorsal nucleus of thalamus
146	Ectorhinal area	Ventral medial nucleus of the thalamus
147	Posterior complex of the thalamus	Retrosplenial area ventral part
148	Ventral anterior-lateral complex of the thalamus	Nucleus of the posterior commissure
149	Dorsal part of the lateral geniculate complex	Parafascicular nucleus
150	Primary auditory area	Culmen
151	Postpiriform transition area	Simple lobule
152	Magnocellular nucleus	Precommissural nucleus
153	Globus pallidus internal segment	Vestibular nuclei
154	Lateral amygdalar nucleus	Parasubiculum
155	Nucleus of the lateral olfactory tract	Ventral posterolateral nucleus of the thalamus
156	Bed nucleus of the accessory olfactory tract	Bed nucleus of the accessory olfactory tract
157	Dorsal premammillary nucleus	Anteroventral preoptic nucleus
158	Substantia nigra reticular part	Subthalamic nucleus
159	Zona incerta	Anterior group of the dorsal thalamus
160	Ventral part of the lateral geniculate complex	Parataenial nucleus
161	Parabigeminal nucleus	Anteroventral periventricular nucleus
162	Field CA3	Postsubiculum
163	Pons	Anterior cingulate area dorsal part
164	Retrochiasmatic area	Secondary motor area
165	Medial amygdalar nucleus	Triangular nucleus of septum
166	Parataenial nucleus	Primary somatosensory area mouth
167	Interanteromedial nucleus of the thalamus	Medial pretectal area
168	Piriform-amygdalar area	Anterior olfactory nucleus
169	Diagonal band nucleus	Primary somatosensory area nose
170	Ventrolateral preoptic nucleus	Anteroventral nucleus of thalamus
171	Anteroventral preoptic nucleus	Periventricular zone
172	Cortical amygdalar area posterior part	Intermediodorsal nucleus of the thalamus
173	Globus pallidus external segment	Medial habenula
174	Posterior limiting nucleus of the thalamus	Anterodorsal nucleus
175	Endopiriform nucleus	Fasciola cinerea
176	Olfactory tubercle	Dorsal peduncular area
177	Central amygdalar nucleus	Induseum griseum
178	Basolateral amygdalar nucleus	Lateral habenula
Number	Methamphetamine hierarchical order	Nicotine hierarchical order
1	Caudoputamen	Ventral tegmental area
2	Anterior amygdalar area	Midbrain reticular nucleus retrorubral area
3	Parataenial nucleus	Superior colliculus motor related
4	Periventricular hypothalamic nucleus preoptic part	Midbrain reticular nucleus
5	Claustrum	Simple lobule
6	Medial habenula	Posterior hypothalamic nucleus
7	Medial pretectal area	Basolateral amygdalar nucleus
8	Ventral part of the lateral geniculate complex	Pedunculopontine nucleus
9	Anteroventral preoptic nucleus	Subparafascicular nucleus
10	Parasubthalamic nucleus	Pons motor related
11	Precommissural nucleus	Anterior cingulate area dorsal part
12	Parastrial nucleus	Paraventricular hypothalamic nucleus
13	Anteroventral periventricular nucleus	Ansiform lobule
14	Central amygdalar nucleus	Presubiculum
15	Lateral amygdalar nucleus	Dorsal auditory area
16	Endopiriform nucleus	Supplemental somatosensory area
17	Paraventricular nucleus of the thalamus	Posterior limiting nucleus of the thalamus
18	Intercalated amygdalar nucleus	Intercalated amygdalar nucleus
19	Intermediodorsal nucleus of the thalamus	Central amygdalar nucleus
20	Postpiriform transition area	Posteromedial visual area
21	Intergeniculate leaflet of the lateral geniculate complex	Lateral visual area
22	Ventral auditory area	Supramammillary nucleus
23	Bed nucleus of the accessory olfactory tract	Anterolateral visual area
24	Basolateral amygdalar nucleus	Gustatory areas
25	Dorsal auditory area	Mammillary body
26	Primary somatosensory area barrel field	Postsubiculum
27	Magnocellular nucleus	Periventricular hypothalamic nucleus posterior part
28	Primary somatosensory area nose	Periventricular zone
29	Induseum griseum	Paraflocculus
30	Anterior cingulate area ventral part	Peripeduncular nucleus
31	Anterior cingulate area dorsal part	Vestibular nuclei
32	Pedunculopontine nucleus	Anteroventral nucleus of thalamus
33	Superior colliculus motor related	Endopiriform nucleus
34	Inferior colliculus	Cortical amygdalar area posterior part
35	Entorhinal area lateral part	Postpiriform transition area
36	Substantia innominata	Prelimbic area
37	Nucleus accumbens	Intermediodorsal nucleus of the thalamus
38	Central lobule	Lateral septal complex
39	Posterior hypothalamic nucleus	Entorhinal area lateral part
40	Substantia nigra compact part	Ventrolateral preoptic nucleus
41	Parabigeminal nucleus	Visceral area
42	Parasubiculum	Posterior auditory area
43	Presubiculum	Temporal association areas
44	Postsubiculum	Primary auditory area
45	Diagonal band nucleus	Ventral auditory area
46	Posterior auditory area	Ectorhinal area
47	Piriform-amygdalar area	Perirhinal area
48	Periaqueductal gray	Pontine reticular nucleus
49	Supramammillary nucleus	Medial geniculate complex
50	Anterolateral visual area	Anterior cingulate area ventral part
51	Primary auditory area	Claustrum
52	Ectorhinal area	Pons
53	Medial geniculate complex	Central lobule
54	Temporal association areas	Red nucleus
55	Perirhinal area	Retrosplenial area lateral agranular part
56	Agranular insular area ventral part	Lateral amygdalar nucleus
57	Paraventricular hypothalamic nucleus	Retrosplenial area dorsal part
58	Subparafascicular nucleus	Interpeduncular nucleus
59	Subparaventricular zone	Superior colliculus sensory related
60	Paraventricular hypothalamic nucleus descending division	Inferior colliculus
61	Nucleus of the brachium of the inferior colliculus	Retrosplenial area ventral part
62	Midbrain reticular nucleus	Periaqueductal gray
63	Anterior hypothalamic nucleus	Ventral part of the lateral geniculate complex
64	Peripeduncular nucleus	Nucleus of the brachium of the inferior colliculus
65	Subiculum	Mediodorsal nucleus of thalamus
66	Lateral visual area	Culmen
67	Superior colliculus sensory related	Fasciola cinerea
68	Midbrain reticular nucleus retrorubral area	Agranular insular area posterior part
69	Nucleus of reuniens	Piriform area
70	Zona incerta	Central medial nucleus of the thalamus
71	Culmen	Interanteromedial nucleus of the thalamus
72	Retrosplenial area lateral agranular part	Medial pretectal area
73	Lateral preoptic area	Thalamus sensory-motor cortex related
74	Anterior pretectal nucleus	Septofimbrial nucleus
75	Posterior limiting nucleus of the thalamus	Ventral posterolateral nucleus of the thalamus
76	Preparasubthalamic nucleus	Piriform-amygdalar area
77	Nucleus of the optic tract	Dorsomedial nucleus of the hypothalamus
78	Medial preoptic area	Dentate gyrus
79	Thalamus sensory-motor cortex related	Anteromedial visual area
80	Medial preoptic nucleus	Posterolateral visual area
81	Dorsomedial nucleus of the hypothalamus	Fundus of striatum
82	Red nucleus	Caudoputamen
83	Lateral septal complex	Arcuate hypothalamic nucleus
84	Central medial nucleus of the thalamus	Parasubthalamic nucleus
85	Interpeduncular nucleus	Suprachiasmatic nucleus
86	Reticular nucleus of the thalamus	Subiculum
87	Medial septal nucleus	Medial septal nucleus
88	Supraoptic nucleus	Nucleus of reuniens
89	Periventricular hypothalamic nucleus posterior part	Substantia nigra compact part
90	Interanteromedial nucleus of the thalamus	Dorsal premammillary nucleus
91	Secondary motor area	Paraventricular hypothalamic nucleus descending division
92	Field CA2	Central lateral nucleus of the thalamus
93	Field CA3	Nucleus of Darkschewitsch
94	Posteromedial visual area	Anterior pretectal nucleus
95	Primary motor area	Parafascicular nucleus
96	Anteromedial visual area	Intergeniculate leaflet of the lateral geniculate complex
97	Medial amygdalar nucleus	Precommissural nucleus
98	Piriform area	Lateral habenula
99	Posterior amygdalar nucleus	Medial habenula
100	Primary somatosensory area trunk	Parabigeminal nucleus
101	Nucleus of the lateral olfactory tract	Nucleus of the optic tract
102	Primary somatosensory area upper limb	Nucleus of the posterior commissure
103	Primary somatosensory area lower limb	Olivary pretectal nucleus
104	Cortical amygdalar area posterior part	Anterodorsal nucleus
105	Visceral area	Posterior pretectal nucleus
106	Agranular insular area posterior part	Parataenial nucleus
107	Gustatory areas	Induseum griseum
108	Supplemental somatosensory area	Triangular nucleus of septum
109	Primary somatosensory area mouth	Paraventricular nucleus of the thalamus
110	Anterior olfactory nucleus	Interanterodorsal nucleus of the thalamus
111	Interanterodorsal nucleus of the thalamus	Medial preoptic area
112	Globus pallidus external segment	Lateral preoptic area
113	Anterodorsal preoptic nucleus	Nucleus accumbens
114	Mediodorsal nucleus of thalamus	Ventral medial nucleus of the thalamus
115	Ventral posterolateral nucleus of the thalamus	Globus pallidus internal segment
116	Median preoptic nucleus	Lateral hypothalamic area
117	Orbital area medial part	Anteroventral periventricular nucleus
118	Infralimbic area	Magnocellular nucleus
119	Prelimbic area	Dorsal peduncular area
120	Taenia tecta	Primary motor area
121	Fundus of striatum	Primary somatosensory area upper limb
122	Lateral habenula	Nucleus of the lateral olfactory tract
123	Olivary pretectal nucleus	Median preoptic nucleus
124	Entorhinal area medial part	Anterodorsal preoptic nucleus
125	Periventricular zone	Primary somatosensory area lower limb
126	Pons	Zona incerta
127	Dorsal premammillary nucleus	Agranular insular area ventral part
128	Pontine reticular nucleus	Field CA3
129	Substantia nigra reticular part	Ventromedial hypothalamic nucleus
130	Lateral hypothalamic area	Parastrial nucleus
131	Ventral tegmental area	Primary visual area
132	Dentate gyrus	Taenia tecta
133	Lateral posterior nucleus of the thalamus	Field CA1
134	Subthalamic nucleus	Field CA2
135	Suprachiasmatic nucleus	Anteroventral preoptic nucleus
136	Posterolateral visual area	Retrochiasmatic area
137	Pons motor related	Infralimbic area
138	Ventromedial hypothalamic nucleus	Anterior amygdalar area
139	Retrochiasmatic area	Primary somatosensory area nose
140	Primary visual area	Submedial nucleus of the thalamus
141	Olfactory tubercle	Primary somatosensory area mouth
142	Retrosplenial area dorsal part	Secondary motor area
143	Field CA1	Subparaventricular zone
144	Mammillary body	Primary somatosensory area trunk
145	Globus pallidus internal segment	Reticular nucleus of the thalamus
146	Arcuate hypothalamic nucleus	Periventricular hypothalamic nucleus preoptic part
147	Ventrolateral preoptic nucleus	Preparasubthalamic nucleus
148	Cuneiform nucleus	Anterior group of the dorsal thalamus
149	Tuberal nucleus	Posterior amygdalar nucleus
150	Submedial nucleus of the thalamus	Tuberal nucleus
151	Dorsal part of the lateral geniculate complex	Paracentral nucleus
152	Retrosplenial area ventral part	Cuneiform nucleus
153	Paraflocculus	Subthalamic nucleus
154	Bed nuclei of the stria terminalis	Substantia nigra reticular part
155	Anteroventral nucleus of thalamus	Entorhinal area medial part
156	Simple lobule	Parasubiculum
157	Fasciola cinerea	Orbital area medial part
158	Dorsal peduncular area	Globus pallidus external segment
159	Triangular nucleus of septum	Olfactory tubercle
160	Orbital area ventrolateral part	Supraoptic nucleus
161	Posterior pretectal nucleus	Dorsal part of the lateral geniculate complex
162	Nucleus of the posterior commissure	Medial preoptic nucleus
163	Nucleus of Darkschewitsch	Posterior complex of the thalamus
164	Frontal pole cerebral cortex	Orbital area lateral part
165	Anterior group of the dorsal thalamus	Ventral anterior-lateral complex of the thalamus
166	Vestibular nuclei	Orbital area ventrolateral part
167	Ventral posterior complex of the thalamus	Lateral dorsal nucleus of thalamus
168	Orbital area lateral part	Substantia innominata
169	Ansiform lobule	Diagonal band nucleus
170	Ventral anterior-lateral complex of the thalamus	Anterior olfactory nucleus
171	Anterodorsal nucleus	Primary somatosensory area barrel field
172	Septofimbrial nucleus	Anterior hypothalamic nucleus
173	Paracentral nucleus	Medial amygdalar nucleus
174	Posterior complex of the thalamus	Bed nuclei of the stria terminalis
175	Ventral medial nucleus of the thalamus	Ventral posterior complex of the thalamus
176	Central lateral nucleus of the thalamus	Frontal pole cerebral cortex
177	Lateral dorsal nucleus of thalamus	Lateral posterior nucleus of the thalamus
178	Parafascicular nucleus	Bed nucleus of the accessory olfactory tract

## Discussion

The present study used unbiased single-cell whole-brain imaging to identify changes in brain functional architecture after withdrawal from chronic exposure to psychostimulants. Withdrawal from psychostimulants resulted in increased functional connectivity that was associated with a decrease in modularity with varying degrees of severity, depending on the drug, compared with control mice. This decreased modularity resulted in the emergence of new network architecture and organization of the brain. Using graph theory, we identified brain regions that are most responsible for intermodular and intramodular communication within each network. Withdrawal from all of the psychostimulants that were tested in the present study resulted in different network organization than the control network. The methamphetamine and cocaine withdrawal networks closely resembled each other in structural organization, primarily through thalamic motifs, whereas the nicotine withdrawal network shared some similarities with the control network. These unbiased whole-brain analyses demonstrate that psychostimulant withdrawal produces the drug-dependent remodeling of functional architecture of the brain and suggest that decreased modularity of the brain functional network may be a central feature of withdrawal.

### Changes to modularity and structure of the brain caused by psychostimulant withdrawal

We found that cocaine, methamphetamine, and nicotine withdrawal produced major increases in functional connectivity throughout the brain compared with control mice. We further found that withdrawal resulted in a decrease in modular structuring of the brain compared with control mice (seven modules). The decrease in modularity was most evident for methamphetamine withdrawal (three modules) and cocaine withdrawal (four modules), whereas nicotine withdrawal showed a smaller reduction of modularity (five modules). Using the same approaches (i.e., whole-brain network analysis of Fos) reduced modularity after abstinence from alcohol dependence in mice was similarly found (Kimbrough et al., 2020). Further, humans who suffer from dementia and traumatic brain injury have shown reduced modularity that is associated with cognitive deficits (de Haan et al., 2012; Brier et al., 2014; Arnemann et al., 2015; Gallen et al., 2016; Sporns and Betzel, 2016; Bertolero et al., 2018). Changes in network structure/functional connectivity (Tomasi et al., 2010; Konova et al., 2013, 2015; Ma et al., 2015) and cognitive function (Spronk et al., 2013; Ashare et al., 2014; Sabrini et al., 2019) have been observed after chronic drug use and withdrawal, suggesting that similar mechanisms may be active between these different neural disorders.

### Features of psychostimulant networks

We examined the components of individual modules within each network and found that the control network was heavily driven by sensory and motor brain regions. This result confers validity to our single-cell whole-brain network analysis approach for characterizing network features because it fits with what might be expected from a normal, awake, behaving animal that explores the environment and relies heavily on sensory/motor systems. Furthermore, the control network was more interconnected between modules overall and contained several regions that could be classified as hubs of each module that are critical for network function, based on high intramodular and intermodular connectivity. This suggests that the control brain may be more resilient to the disruption of function because additional hub regions may compensate more easily in response to such disruptions.

In the networks that were associated with withdrawal from psychostimulants, a shift was observed from sensory/motor regions to more subcortical (e.g., amygdalar, thalamic, hypothalamic, and midbrain) regions that drive the network. A similar effect was seen in nonhuman primates after cocaine abstinence (Murnane et al., 2015), and alterations of functional connectivity of the somatosensory cortex are associated with smokers (Claus and Weywadt, 2020). This may represent a shift from top-down cortical network control (Gilbert and Sigman, 2007) to bottom-up subcortical network control and may reflect the greater influence of internal drives that are associated with negative affect during withdrawal in controlling the whole-brain network (Koob, 2015). This shift may be a major reason why drugs are so addictive because higher cortical functional connectivity in humans may protect against relapse (McHugh et al., 2017).

Given the modular organization of the different networks, both the control network and nicotine network had a much higher incidence of intermodular connectivity, whereas the methamphetamine and cocaine networks had only a small subset of brain regions that were connected between different modules. Similar changes in neural activity, combined with decreases in interconnectivity and network efficiency, have been observed in humans after psychostimulant use (Ahmadlou et al., 2013; Wang et al., 2015; Liang et al., 2015). The nicotine network was different from the methamphetamine and cocaine networks and somewhat resembled a slightly altered control network. Similarities and differences in network properties of the three different drugs are likely to be caused by differences in receptor mechanisms and locations where each drug acts throughout the brain. Indeed, both cocaine and methamphetamine target the same dopamine transporter, whereas nicotine acts on nicotinic receptors (Rothman and Baumann, 2003; Nestler, 2005; Sulzer et al., 2005; D’Souza and Markou, 2011).

The interanterodorsal nucleus of the thalamus was disconnected from the nicotine network, suggesting that it may not be involved in controlling the withdrawal network, although we cannot exclude the possibility that its disconnection may instead be a critical feature of nicotine withdrawal. One of the larger modules in the nicotine network was driven by several brain regions, two of which included the AAA and AIv, which have been suggested to be associated with nicotine withdrawal in humans (Naqvi et al., 2007; Sutherland et al., 2013). The methamphetamine and cocaine networks, although having distinctly different features, shared an overall motif of lower modularity and being heavily driven by thalamic brain regions. This suggests that, in a destabilized and less structured neural network, the thalamus becomes more critical to controlling the whole-brain network. The thalamus is thought to play a major role in relaying information, and the reliance of these networks on this group of regions suggests that the thalamus is not simply a relay station but has greater importance in cognitive and emotional function (Sherman, 2007; Ahissar and Oram, 2015). Substantial evidence corroborates the importance of the thalamus in psychostimulant addiction and withdrawal. In a rat model of cocaine self-administration, the thalamus was found to be heavily involved in network function during acute abstinence, but changes in the network disappeared after two weeks (Orsini et al., 2018). Interestingly, the thalamus in humans has been shown to be hypoactive in cocaine abusers (Tomasi et al., 2007), and thalamic connectivity is predictive of cocaine dependence (Zhang et al., 2016) and altered in infants who are exposed to cocaine (Salzwedel et al., 2016). Although network changes that are induced by acute withdrawal are reversed over time (Orsini et al., 2018), prolonged use may lead to more permanent restructuring of the brain, and major differences between the nicotine and methamphetamine/cocaine networks may account for differences in the severity of each drug after long-term use (Nestler, 2005; Grant et al., 2012; Spronk et al., 2013).

In conclusion, in the past 40 years, the substance use disorder field has made tremendous progress by identifying numerous brain regions that are dysregulated after psychostimulant exposure and contribute to withdrawal behaviors (Kalivas and McFarland, 2003; Robinson and Kolb, 2004; Kalivas, 2007; Everitt et al., 2008; Jedynak et al., 2012; Koob and Volkow, 2016; Bobadilla et al., 2017). The present results confirm that a substantial number of brain regions are affected by psychostimulant exposure and suggest that a common pathway that is associated with withdrawal may not reside at the level of brain regions or even single neural circuits. Instead, these results suggest that the main common phenomenon that is observed among all three of these psychostimulants is decreased modularity of whole-brain functional architecture, suggesting that a common feature may reside at the whole-network level. This interpretation is consistent with the literature on the modularity of complex systems, including the brain and mind, showing that lower modularity reduces the capacity of the system to adapt to its environment (Kashtan and Alon, 2005). It is however worth noting that further studies will be necessary to determine whether lower modularity is simply a feature of increased functional connectivity regardless of whether it is because of withdrawal or other mechanisms. One limitation of the present study is that it did not assess withdrawal behaviors after minipump removal for comparison to network changes. This was done to avoid confounds as to the source of Fos production (e.g., withdrawal or behavioral testing). Another limitation of the present study is the lack of direct comparisons between neural activation of each treatment. The approaches used within this study can be leveraged to study and better understand numerous cognitive states (Smith and Kimbrough, 2020; Simpson et al., 2021). However, in the future assessing neural and network differences in more quantitative ways will be necessary.

In summary, the present study showed that withdrawal from psychostimulants results in changes in neural network structure, including increases in functional connectivity among brain regions and decreases in modularity. Psychostimulant withdrawal resulted in a shift from a sensory/motor-driven network to a network that is highly driven by subcortical regions. We also found that different psychostimulants do not produce the same neural networks, although methamphetamine and cocaine shared similar properties. These findings shed light on alterations of brain function that are caused by drug exposure and identify potential brain regions that warrant future study. The present study demonstrates that psychostimulant withdrawal produces drug-dependent remodeling of the functional architecture of the brain and suggests that decreased modularity of the brain functional networks may be a common feature of withdrawal. These findings may prove critical to designing future treatment approaches for withdrawal symptoms.

## Synthesis

Reviewing Editor: Yavin Shaham, NIDA-IRP/NIH Decisions are customarily a result of the Reviewing Editor and the peer reviewers coming together and discussing their recommendations until a consensus is reached. When revisions are invited, a fact-based synthesis statement explaining their decision and outlining what is needed to prepare a revision will be listed below. The following reviewer(s) agreed to reveal their identity: NONE. Note: If this manuscript was transferred from JNeurosci and a decision was made to accept the manuscript without peer review, a brief statement to this effect will instead be what is listed below.

The authors satisfactory addressed many of the comments of the reviewers but one of the reviewers continue to raise significant technical concerns that should be addressed in the revision. Note that while reviewer 1 was initially very positive, after the editorial consultations the reviewer agreed that the technical concerns of reviewer 2 are valid and should be addressed in the revision. In the revision, we recommend moving most supplemental figures to the main text, and also include the new validation figures in the main text. I enclose the original reviewers’ comments below.

REVIEWER 1

The authors have addressed all my concerns by providing the requested supplementary data and by limiting the discussion to drug withdrawal. I look forward to future studies testing whether (and how) the identified network shifts affect drug-seeking.

REVIEWER 2

I apologize for the negative comments that follow, but I feel more technical questions have been raised by the inclusion of the new Extended Figure 1 than have been answered. I am uncomfortable reviewing the major biological findings of the manuscript until the technical concerns have been addressed properly. I believed this would be easy for the authors to accomplish, since such validation of their staining, imagining, and analysis must have been performed, but it is not shown in the resubmission.

Specifically, my technical concerns are not alleviated by the data shown in the resubmission. Because of the omission of raw data in the initial submission, my main critique was to include raw data in the resubmission, along with some validation of cell segmentation and brain registration. Since the graph theory analysis, which is the cornerstone of this manuscript, depends on accurate cell segmentation and brain registration, the continued omission of detailed raw data or validation is troubling.

Specifically, I asked: “ Representative IHC planes from the imaged volumes, both zoomed out for the whole plane and insets with areas of interest, of regions with significant differences between groups that are used in further analysis.”

In response, Extended Figure 1 includes low-resolution insets from a single plane within the hippocampus. These representative images raise more questions and concerns than they answer. The comparison I will draw is to papers using standard Fos IHC, where at a minimum I would ask for clear representative images from each condition. With an approach like whole brain imaging, there is an even greater need for such images and validation experiments. How consistent is staining throughout the whole volume? How consistent is imaging throughout the whole volume? How were the parameters for automated ClearMap segmentation validated?

I am surprised that such images are not being highlighted in this manuscript, like most other whole brain papers, so that readers can appreciate the scope of the data provided by this method!

These images should be easy to produce, as the full analysis is based on them. It is troubling that they are not. At the very least Extended Figure 1 should be included as a main figure so that readers of the manuscript can judge the quality of whole brain staining and imaging within the context of the more computational analysis provided. It should not be hidden in the Extended Figures.

Again, until the technical concerns are alleviated I will not comment on the interpretation of the data.
