# Supplementary figures and images for: Characterization of the Brain Functional Architecture of Psychostimulant Withdrawal Using Single-Cell Whole-Brain Imaging
Source: eNeuro. 2021 Nov 2;8(6):ENEURO.0208-19.2021. doi: 10.1523/ENEURO.0208-19.2021 (PMC8570684; doi:10.1523/ENEURO.0208-19.2021)

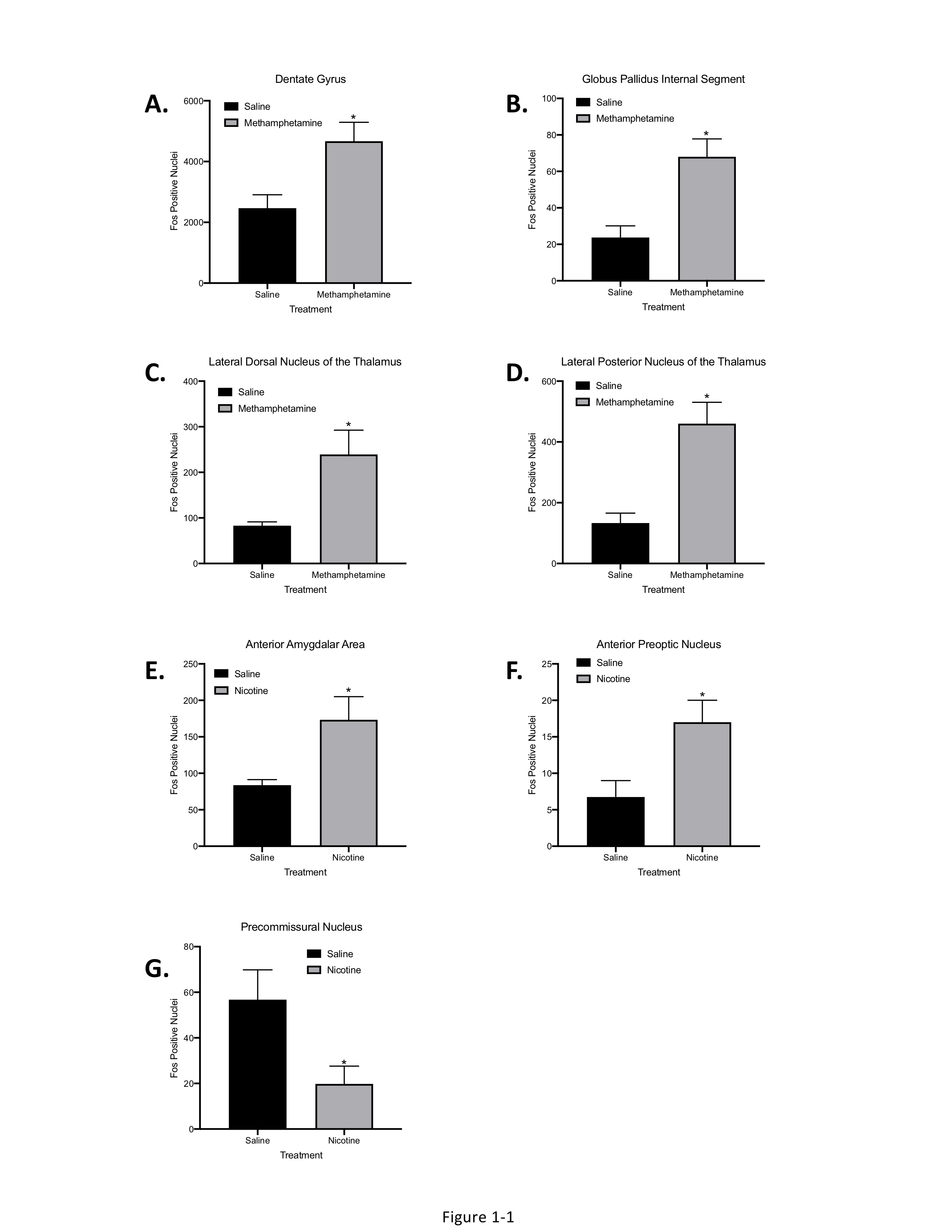

Supplement: Extended Data Figure 2-1 — Fos counts of brain regions showing significant differences between a treatment and saline. Download Figure 2-1, TIF file. [file enu-eN-NWR-0208-19-s01.tif]

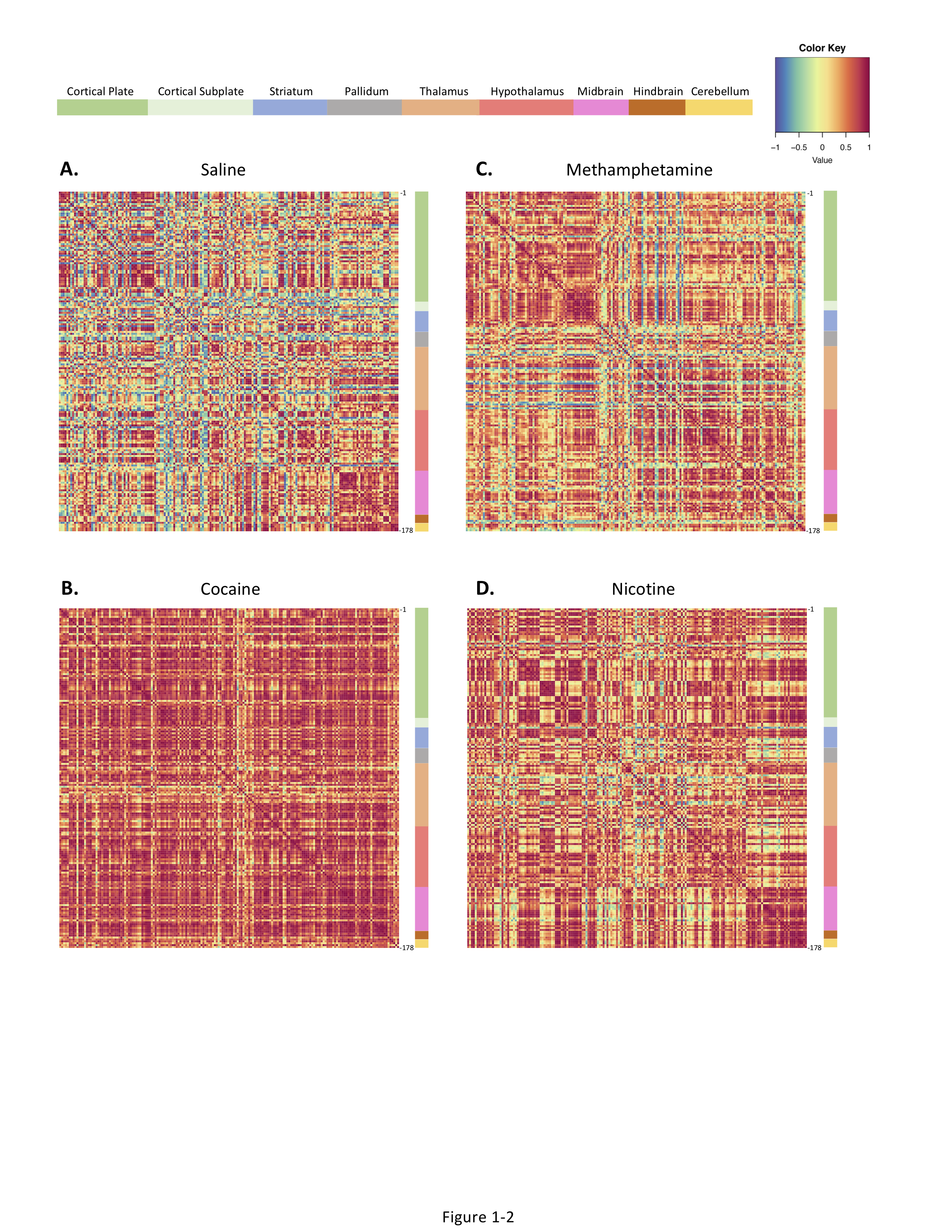

Supplement: Extended Data Figure 3-1 — Pearson correlation matrices for showing functional connectivity measures of each treatment. Download Figure 3-1, TIF file. [file enu-eN-NWR-0208-19-s02.tif]
